# Effects of Probiotics, Prebiotics, and Synbiotics on Human Health

**DOI:** 10.3390/nu9091021

**Published:** 2017-09-15

**Authors:** Paulina Markowiak, Katarzyna Śliżewska

**Affiliations:** Institute of Fermentation Technology and Microbiology, Department of Biotechnology and Food Sciences, Lodz University of Technology, 90-924 Łódź, Poland

**Keywords:** probiotic bacteria, prebiotics, synbiotics, human health, gut microbiota

## Abstract

The human gastrointestinal tract is colonised by a complex ecosystem of microorganisms. Intestinal bacteria are not only commensal, but they also undergo a synbiotic co-evolution along with their host. Beneficial intestinal bacteria have numerous and important functions, e.g., they produce various nutrients for their host, prevent infections caused by intestinal pathogens, and modulate a normal immunological response. Therefore, modification of the intestinal microbiota in order to achieve, restore, and maintain favourable balance in the ecosystem, and the activity of microorganisms present in the gastrointestinal tract is necessary for the improved health condition of the host. The introduction of probiotics, prebiotics, or synbiotics into human diet is favourable for the intestinal microbiota. They may be consumed in the form of raw vegetables and fruit, fermented pickles, or dairy products. Another source may be pharmaceutical formulas and functional food. This paper provides a review of available information and summarises the current knowledge on the effects of probiotics, prebiotics, and synbiotics on human health. The mechanism of beneficial action of those substances is discussed, and verified study results proving their efficacy in human nutrition are presented.

## 1. Introduction

Nowadays, besides the basic role of nutrition consisting in the supply of necessary nutrients for growth and development of the organism, some additional aspects are becoming increasingly important, including the maintenance of health and counteracting diseases. In the world of highly processed food, particular attention is drawn to the composition and safety of consumed products. The quality of food is very important because of, i.e., the problem of food poisoning, obesity, allergy, cardiovascular diseases, and cancer—the plague of the 21st century. Scientific reports point to the health benefits of using probiotics and prebiotics in human nutrition. The word “probiotic” comes from Greek, and it means “for life”. Most probably, it was Ferdinand Vergin who invented the term “probiotic” in 1954, in his article entitled “Anti-und Probiotika” comparing the harmful effects of antibiotics and other antibacterial agents on the intestinal microbiota with the beneficial effects (“probiotika”) of some useful bacteria [1]. Some time after that, in 1965, Lilly and Stillwell described probiotics as microorganisms stimulating the growth of other microorganisms [2]. The definition of probiotics has been modified and changed many times. To emphasise their microbial origin, Fuller (1989) stated that probiotics must be viable microorganisms and must exert a beneficial effect on their host [3]. On the other hand, Guarner and Schaafsma (1998) indicated the necessary use of an appropriate dose of probiotic organisms required to achieve the expected effect [4]. The current definition, formulated in 2002 by FAO (Food and Agriculture Organization of the United Nations) and WHO (World Health Organization) working group experts, states that probiotics are “live strains of strictly selected microorganisms which, when administered in adequate amounts, confer a health benefit on the host” [5]. The definition was maintained by the International Scientific Association for Probiotics and Prebiotics (ISAPP) in 2013 [6].

Results of clinical studies confirm the positive effect of probiotics on gastrointestinal diseases (e.g., irritable bowel syndrome, gastrointestinal disorders, elimination of *Helicobacter*, inflammatory bowel disease, diarrhoeas) and allergic diseases (e.g., atopic dermatitis). Many clinical studies have proven the effectiveness of probiotics for treatment of diseases such as obesity, insulin resistance syndrome, type 2 diabetes, and non-alcoholic fatty liver disease. Furthermore, the positive effects of probiotics on human health have been demonstrated by increasing the body’s immunity (immunomodulation). Scientific reports also show the benefits of the prophylactic use of probiotics in different types of cancer and side effects associated with cancer. Many clinical studies have proven the effectiveness of probiotics, and recommended doses of probiotics are those that have been used in a particular case. Keep in mind that how probiotics work may depend on the strain, dose, and components used to produce a given probiotic product.

In 1995, prebiotics were defined by Gibson and Roberfroid as non-digested food components that, through the stimulation of growth and/or activity of a single type or a limited amount of microorganisms residing in the gastrointestinal tract, improve the health condition of a host [7]. In 2004, the definition was updated and prebiotics were defined as selectively fermented components allowing specific changes in the composition and/or activity of microorganisms in the gastrointestinal tract, beneficial for host’s health and wellbeing [8]. Finally, in 2007, FAO/WHO experts described prebiotics as a nonviable food component that confers a health benefit on the host associated with modulation of the microbiota [9].

Prebiotics may be used as an alternative to probiotics or as an additional support for them. However different prebiotics will stimulate the growth of different indigenous gut bacteria. Prebiotics have enormous potential for modifying the gut microbiota, but these modifications occur at the level of individual strains and species and are not easily predicted a priori. There are many reports on the beneficial effects of prebiotics on human health.

High potential is attributed to the simultaneous use of probiotics and prebiotics. In 1995, Gibson and Roberfroid introduced the term “synbiotic” to describe a combination of synergistically acting probiotics and prebiotics [7]. A selected component introduced to the gastrointestinal tract should selectively stimulate growth and/or activate the metabolism of a physiological intestinal microbiota, thus conferring beneficial effect to the host’s health [10]. As the word “synbiotic” implies synergy, the term should be reserved for those products in which a prebiotic component selectively favours a probiotic microorganism [11]. The principal purpose of that type of combination is the improvement of survival of probiotic microorganisms in the gastrointestinal tract.

Synbiotics have both probiotic and prebiotic properties and were created in order to overcome some possible difficulties in the survival of probiotics in the gastrointestinal tract [12]. Therefore, an appropriate combination of both components in a single product should ensure a superior effect, compared to the activity of the probiotic or prebiotic alone [13,14].

The aim of the review was to discuss the mechanisms of action of probiotics, prebiotics, and synbiotics, as well as the current insight into their effect on human health. The selection of probiotic strains, prebiotics, and their respective dosages is crucial in obtaining a therapeutic effect, so separate sections are dedicated to this topic. Further research into the acquisition of new probiotic strains, the selection of probiotics and prebiotics for synbiotics, dose setting, safety of use, and clinical trials documenting the desired health effects is necessary. Effects should be confirmed in properly scheduled clinical trials conducted by independent research centres.

## 2. Probiotics

The knowledge of the beneficial effects of lactic acid fermentation on human health dates back to ancient times. The Bible mentions sour milk several times. Ancient Romans and Greeks knew various recipes for fermented milk. A specific type of sour milk, called “leben raib”, prepared from buffalo, cow, or goat milk, was consumed in ancient Egypt. A similar “jahurt” was also commonly consumed by people inhabiting the Balkans. In India, fermented milk drinks were known already 800–300 years B.C., and in Turkey in the 8th century. A milk drink called “ajran” was consumed in Central Russia in the 12th century, and “tarho” was consumed in Hungary in the 14th century [15].

A particular interest in lactic acid fermentation was expressed in the beginning of the 20th century by the Russian scientist and immunologist working for the Pasteur Institute in Paris, awarded with the Nobel Prize in medicine for his work on immunology (in 1907), Ilia Miecznikow. Here is a quote from his book “Studies on Optimism”: “with various foods undergoing lactic acid fermentation and consumed raw (sour milk, kefir, sauerkraut, pickles) humans introduced huge amounts of proliferating lactic acid bacteria to their alimentary tracts” [16].

### 2.1. Selection Criteria and Requirements for Probiotic Strains

According to the suggestions of the WHO, FAO, and EFSA (the European Food Safety Authority), in their selection process, probiotic strains must meet both safety and functionality criteria, as well as those related to their technological usefulness (Table 1). Probiotic characteristics are not associated with the genus or species of a microorganism, but with few and specially selected strains of a particular species [6]. The safety of a strain is defined by its origin, the absence of association with pathogenic cultures, and the antibiotic resistance profile. Functional aspects define their survival in the gastrointestinal tract and their immunomodulatory effect. Probiotic strains have to meet the requirements associated with the technology of their production, which means they have to be able to survive and maintain their properties throughout the storage and distribution processes [17]. Probiotics should also have documented pro-health effects consistent with the characteristics of the strain present in a marketed product. Review papers and scientific studies on one strain may not be used for the promotion of other strains as probiotics. It has to be considered, as well, that the studies documenting probiotic properties of a particular strain at a tested dose do not constitute evidence of similar properties of a different dose of the same strain. Also, the type of carrier/matrix is important, as it may reduce the viability of a particular strain, thus changing the properties of a product [18,19].

### 2.2. Probiotic Microorganisms

Probiotic products may contain one or more selected microbial strains. Human probiotic microorganisms belong mostly to the following geni: *Lactobacillus*, *Bifidobacterium*, and *Lactococus*, *Streptococcus*, *Enterococcus*. Moreover, strains of Gram-positive bacteria belonging to the genus *Bacillus* and some yeast strains belonging to the genus *Saccharomyces* are commonly used in probiotic products [21].

Probiotics are subject to regulations contained in the general food law, according to which they should be safe for human and animal health. In the USA, microorganisms used for consumption purposes should have the GRAS (Generally Regarded As Safe) status, regulated by the FDA (Food and Drug Administration). In Europe, EFSA introduced the term of QPS (Qualified Presumption of Safety). The QPS concept involves some additional criteria of the safety assessment of bacterial supplements, including the history of safe usage and absence of the risk of acquired resistance to antibiotics [22,23]. Table 2 presents probiotic microorganisms contained in pharmaceutical products and as food additives.

### 2.3. Mechanism of Action of Probiotics

A significant progress has been observed lately in the field of studies on probiotics, mostly in terms of the selection and characteristics of individual probiotic cultures, their possible use, and their effect on health.

Probiotics have numerous advantageous functions in human organisms. Their main advantage is the effect on the development of the microbiota inhabiting the organism in the way ensuring proper balance between pathogens and the bacteria that are necessary for a normal function of the organism [27,28]. Live microorganisms meeting the applicable criteria are used in the production of functional food and in the preservation of food products. Their positive effect is used for the restoration of natural microbiota after antibiotic therapy [29,30]. Another function is counteracting the activity of pathogenic intestinal microbiota, introduced from contaminated food and environment. Therefore, probiotics may effectively inhibit the development of pathogenic bacteria, such as *Clostridium perfringens* [31], *Campylobacter jejuni* [32], *Salmonella* Enteritidis [33], *Escherichia coli* [34], various species of *Shigella* [35], *Staphylococcus* [36], and *Yersinia* [37], thus preventing food poisoning. A positive effect of probiotics on digestion processes, treatment of food allergies [38,39], candidoses [40], and dental caries [41] has been confirmed. Probiotic microorganisms such as *Lactobacillus plantarum* [42], *Lactobacillus reuteri* [43], *Bifidobacterium adolescentis*, and *Bifidobacterium pseudocatenulatum* [44] are natural producers of B group vitamins (B1, B2, B3, B6, B8, B9, B12). They also increase the efficiency of the immunological system, enhance the absorption of vitamins and mineral compounds, and stimulate the generation of organic acids and amino acids [18,45,46,47]. Probiotic microorganisms may also be able to produce enzymes, such as esterase, lipase, and co-enzymes A, Q, NAD, and NADP. Some products of probiotics’ metabolism may also show antibiotic (acidophiline, bacitracin, lactacin), anti-cancerogenic, and immunosuppressive properties [45,48,49,50].

Molecular and genetic studies allowed the determination of the basics of the beneficial effect of probiotics, involving four mechanisms:

(1) Antagonism through the production of antimicrobial substances [51];
(2) Competition with pathogens for adhesion to the epithelium and for nutrients [52];
(3) Immunomodulation of the host [53];
(4) Inhibition of bacterial toxin production [54].

The first two mechanisms are directly associated with their effect on other microorganisms. Those mechanisms are important in prophylaxis and treatment of infections, and in the maintenance of balance of the host’s intestinal microbiota. The ability of probiotic strains to co-aggregate, as one of their mechanisms of action, may lead to the formation of a protective barrier preventing pathogenic bacteria from the colonisation of the epithelium [27]. Probiotic bacteria may be able to adhere to epithelial cells, thus blocking pathogens. That mechanism exerts an important effect on the host’s health condition. Moreover, the adhesion of probiotic microorganisms to epithelial cells may trigger a signalling cascade, leading to immunological modulation. Alternatively, the release of some soluble components may cause a direct or indirect (through epithelial cells) activation of immunological cells. This effect plays an important role in the prevention and treatment of contagious diseases, as well as in chronic inflammation of the alimentary tract or of a part thereof [28]. There are also suggestions of a possible role of probiotics in the elimination of cancer cells [55].

Results of in vitro studies indicate the role of low-molecular-weight substances produced by probiotic microorganisms (e.g., hydroperoxide and short-chain fatty acids) in inhibiting the replication of pathogens [28]. For example, *Lactobacillus* genus bacteria may be able to produce bacteriocins, including low-molecular-weight substances (LMWB—antibacterial peptides), as well as high-molecular-weight ones (class III bacteriocins), and some antibiotics. Probiotic bacteria (e.g., *Lactobacillus* and *Bifidobacterium*) may produce the so-called de-conjugated bile acids (derivatives of bile acids), demonstrating stronger antibacterial effect than the bile salts produced by their host [28,56]. Further studies are necessary to explain the mechanism of acquiring resistance to their own metabolites by *Lactobacillus* genus bacteria. The nutrient essential for nearly all bacteria, except for lactic acid bacteria, is iron. It turns out that *Lactobacillus* bacteria do not need iron in their natural environment, which may be their crucial advantage over other microorganisms [57]. *Lactobacillus delbrueckii* affects the function of other microbes by binding iron hydroxide to its cellular surface, thus making it unavailable to other microbes [58].

The immunomodulatory effect of the intestinal microbiota, including probiotic bacteria, is based on three, seemingly contradictory phenomena [53,59]:

(1) Induction and maintenance of the state of immunological tolerance to environmental antigens (nutritional and inhalatory);
(2) Induction and control of immunological reactions against pathogens of bacterial and viral origin;
(3) Inhibition of auto-aggressive and allergic reactions.

Probiotic-induced immunological stimulation is also manifested by the increased production of immunoglobulins, enhanced activity of macrophages and lymphocytes, and stimulation of γ-interferon production. Probiotics may influence the congenital and acquired immunological system through metabolites, components of the cellular wall, and DNA, recognised by specialised cells of the host (e.g., those equipped with receptors) [28]. The principal host cells that are important in the context of the immune response are intestinal epithelial cells and intestinal immune cells. Components of the cellular wall of lactic acid bacteria stimulate the activity of macrophages. Those, in turn, are able to destroy microbes rapidly by the increased production of free oxygen radicals and lysosomal enzymes. Probiotic bacteria are also able to stimulate the production of cytokines by immunocompetent cells of the gastrointestinal tract [60]. On the other hand, the immunological activity of yeast is associated with the presence of glucans in their cellular wall. Those compounds stimulate the response of the reticuloendothelial system [61].

The last of the abovementioned probiotic effects—inhibition of the production of bacterial toxins—is based on actions leading to toxin inactivation and help with the removal of toxins from the body. Help in detoxification from the body can take place by adsorption (some strains can bind toxins to their cell wall and reduce the intestinal absorption of toxins), but can also result from the metabolism of mycotoxins (e.g., aflatoxin) by microorganisms [62,63,64]. However, not all probiotics exhibit detoxifying properties, as it is a strain-related characteristic. Studies should therefore be conducted to select strains with such characteristics. The effectiveness of some probiotics in combating diarrhoea is probably associated with their ability to protect the host from toxins. The reduction of metabolic reactions leading to the production of toxins is also associated with the stimulation of pathways leading to the production of native enzymes, vitamins, and antimicrobial substances [28].

Gut microbiota play a significant role in host metabolic processes (e.g., the regulation of cholesterol absorption, blood pressure (BP), and glucose metabolism), and recent metagenomic surveys have revealed that they are involved in host immune modulation and that they influence host development and physiology (organ development) [65,66,67]. Nutritional programming to manipulate the composition of the intestinal microbiota through the administration of probiotics continues to receive much attention for the prevention or attenuation of the symptoms of metabolic-related diseases. Currently, studies are exploring the potential for expanded uses of probiotics for improving health conditions in metabolic disorders that increase the risk of developing cardiovascular diseases such as hypertension. Further investigations are required to evaluate the targeted and effective use of the wide variety of probiotic strains in various metabolic disorders to improve the overall health status of the host [65].

**Figure 1:**
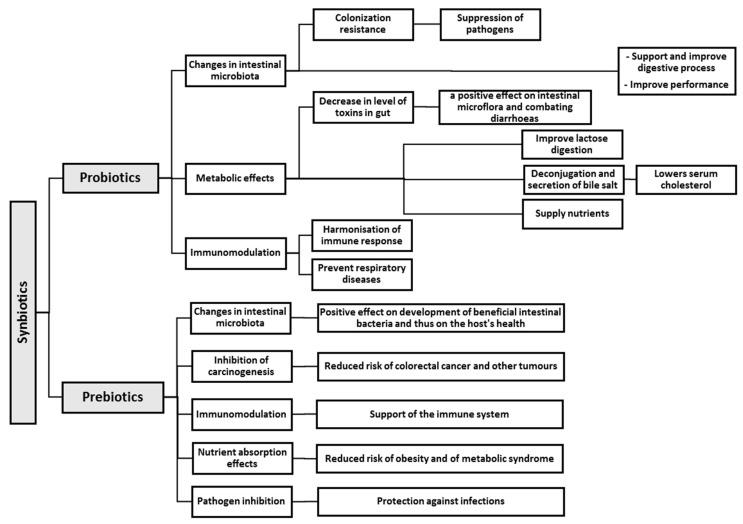
Mechanisms of action of synbiotics and their effects.

In order to confirm the beneficial role of probiotics in improving cardiovascular health and in the reduction of BP, more extensive studies are needed to understand the mechanisms underlying probiotic action. Most probably, all of the abovementioned mechanisms of probiotic action have an effect on the protection against infections, cancer, and the stabilization of balance of the host’s intestinal microbiota. However, it seems unlikely that each of the probiotic microorganisms has properties of all four aspects simultaneously and constitutes a universal remedy to multiple diseases. An important role in the action of probiotics is played by species- and strain-specific traits, such as: cellular structure, cell surface, size, metabolic properties, and substances secreted by microorganisms. The use of a combination of probiotics demonstrating various mechanisms of action may provide enhanced protection offered by a bio-therapeutic product [68]. Figure 1 summarises the mechanisms and effects of action of probiotics.

### 2.4. Probiotics for Humans

In the face of widespread diseases and ageing societies, the use of knowledge on microbiocenosis of the gastrointestinal tract and on the beneficial effect of probiotic bacteria is becoming increasingly important. The consumption of pre-processed food (fast food), often containing excessive amounts of fat and insufficient amounts of vegetables, is another factor of harmful modification of human intestinal microbiota. There is currently no doubt about the fact that the system of intestinal microorganisms and its desirable modification with probiotic formulas and products may protect people against enteral problems, and influence the overall improvement of health.

Probiotics may be helpful in the treatment of inflammatory enteral conditions, including ulcerative colitis, Crohn’s disease, and non-specific ileitis. The aetiology of those diseases is not completely understood, but it is evident that they are associated with chronic and recurrent infections or inflammations of the intestine. Clinical studies have demonstrated that probiotics lead to the remission of ulcerative colitis, but no positive effect on Crohn’s disease has been observed [69,70]. Numerous studies assessed the use of probiotics in the treatment of lactose intolerance [71,72], irritable bowel syndrome, and the prevention of colorectal cancer [73] and peptic ulcers [74].

Considering their role in the inhibition of some bacterial enzymes, probiotics may reduce the risk of colorectal carcinoma in animals. However, the same effect in humans has not been confirmed in clinical trials [75]. On the other hand, a positive effect on the urogenital system (prevention and treatment of Urinary Tract Infections (UTIs) and bacterial vaginitis) constitutes an excellent example of the benefits associated with the use of probiotics [76,77,78]. There were attempts to apply probiotics to pregnant women and neonates in order to prevent allergic diseases such as atopic dermatitis. However, the scope of action is controversial in this kind of case [79]. There is evidence that the consumption of probiotics-containing dairy products results in the reduction of blood cholesterol, which may be helpful in the prevention of obesity, diabetes, cardiovascular diseases, and cerebral stroke [80]. The reduction of cholesterol level achieved due to probiotics is less pronounced compared to the effect of pharmaceutical agents, but leads to a significant minimisation of side effects [80]. Other studies confirmed the effect of the probiotic formula VSL#3 and of the *Oxalobacter formigenes* bacterial strain on the elimination of oxalates with urine, which may potentially reduce the risk of urolithiasis [81]. Studies on animals demonstrated that orally administered *Lactobacillus acidophilus* induces expression of *μ*-opioid and cannabinoid receptors in intestinal cells and mediate analgesic functions in the intestine, and that the observed effect is comparable to the effect of morphine [82]. However, the effect has not been demonstrated in humans.

There are many reports on the application of probiotics in the treatment of diarrhoea. The application of *Saccharomyces boulardii* yeast to patients with acute, watery diarrhoea resulted in the cure and reduced frequency of that type of complaints in two subsequent months [83]. The efficacy of probiotic strains in the therapy of nosocomial, non-nosocomial, and viral diarrhoeas has also been documented. It turns out that probiotics may increase the amount of IgA antibodies, which leads to the arrest of a viral infection [84].

Antibiotic-associated diarrhoea (AAD) is a common complication of most antibiotics and *Clostridium difficile* disease (CDD), which also is incited by antibiotics, and is a leading cause of nosocomial outbreaks of diarrhoea and colitis. The use of probiotics for these two related diseases remains controversial. A variety of different types of probiotics show promise as effective therapies for these two diseases. Using meta-analyses, three types of probiotics (*Saccharomyces boulardii*, *Lactobacillus rhamnosus* GG, and probiotic mixtures) significantly reduced the development of antibiotic-associated diarrhoea. Only *S. boulardii* was effective for CDD [85].

Studies performed in a foster home in Helsinki (Finland) demonstrated that the regular use of *Lactobacillus rhamnosus* GG in the form of a probiotic resulted in a reduced number of respiratory tract infections [86]. Other studies demonstrated that the application of a diet depleted of fermented foods caused a reduction of congenital immunological response, as well as a significant reduction of stool *Lactobacillus* count and of the stool amount of short-chain fatty acids. Moreover, the reduction of phagocytic activity of leukocytes was observed after two weeks of the diet, which could have a negative impact on the organism’s ability to protect against infections [87]. The effect of a fermented product containing *Lactobacillus gasseri* CECT5714 and *Lactobacillus coryniformis* CECT5711 strains on blood and stool parameters was studied in a randomised, double-blind trial on 30 healthy volunteers. No negative effects were observed in the group of subjects receiving the probiotic strains. Some positive effects were observed, including: the production of short-chain fatty acids, humidity, frequency and volume of stools, and subjective improvement of intestinal function [88]. Studies by Alvaro et al. (2007) demonstrated a significant reduction of *Enterobacteriaceae* count and increased galactosidase activity in the alimentary tract of yoghurt consumers, compared to those who did not eat yoghurt [89]. Table 3 lists the results of studies focusing on the effect of probiotics on human health. There are examples of clinical trials during which the probiotics group received the probiotic prophylactically or in addition to the standard therapy.

## 3. Prebiotics

Different prebiotics will stimulate the growth of different indigenous gut bacteria. Prebiotics have enormous potential for modifying the gut microbiota, but these modifications occur at the level of individual strains and species and are not easily predicted a priori. Furthermore, the gut environment, especially pH, plays a key role in determining the outcome of interspecies competition. Both for reasons of efficacy and of safety, the development of prebiotics intended to benefit human health has to take account of the highly individual species profiles that may result [129].

Fruit, vegetables, cereals, and other edible plants are sources of carbohydrates constituting potential prebiotics. The following may be mentioned as such potential souces: tomatoes, artichokes, bananas, asparagus, berries, garlic, onions, chicory, green vegetables, legumes, as well as oats, linseed, barley, and wheat [130]. Some artificially produced prebiotics are, among others: lactulose, galactooligosaccharides, fructooligosaccharides, maltooligosaccharides, cyclodextrins, and lactosaccharose. Lactulose constitutes a significant part of produced oligosaccharides (as much as 40%). Fructans, such as inulin and oligofructose, are believed to be the most used and effective in relation to many species of probiotics [131].

### 3.1. Prebiotic Selection Criteria

**Figure 2:**
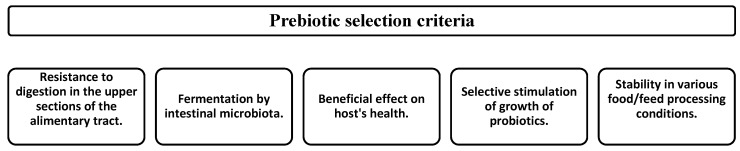
Requirements for potential prebiotics [132,138].

According to Wang (2009), there are five basic criteria for the classification of food components such as prebiotics (Figure 2) [132]. The first criterion assumes that prebiotics are not digested (or just partially digested) in the upper segments of the alimentary tract. As a consequence, they reach the colon, where they are selectively fermented by potentially beneficial bacteria (a requirement of the second criterion) [133]. The fermentation may lead to the increased production or a change in the relative abundance of different short-chain fatty acids (SCFAs), increased stool mass, a moderate reduction of colonic pH, reduction of nitrous end products and faecal enzymes, and an improvement of the immunological system [134], which is beneficial for the host (the requirement of the third criterion). Selective stimulation of growth and/or activity of the intestinal bacteria potentially associated with health protection and wellbeing is considered another criterion [8]. The last criterion of the classification assumes that a prebiotic must be able to withstand food processing conditions and remained unchanged, non-degraded, or chemically unaltered and available for bacterial metabolism in the intestine [132]. Huebner et al. (2008) tested several commercially available prebiotics using various processing conditions. They found no significant changes of the prebiotic activity of the tested substances in various processing conditions [135]. Meanwhile, Ze et al. (2012) showed that it was possible to alter the ability of gut bacteria by utilising starch in vitro [136]. The structure of prebiotics should be appropriately documented, and components used as pharmaceutical formulas, food, or feed additives should be relatively easy to obtain at an industrial scale [137].

Prebiotics may be used as an alternative to probiotics or as an additional support for them. Long-term stability during the shelf-life of food, drinks, and feed, resistance to processing, and physical and chemical properties that exhibit a positive effect on the flavour and consistence of products may promote prebiotics as a competition to probiotics. Additionally, resistance to acids, proteases, and bile salts present in the gastrointestinal tract may be considered as other favourable properties of prebiotics. Prebiotic substances selectively stimulate microorganisms present in the host’s intestinal ecosystem, thus eliminating the need for competition with bacteria. Stimulation of the intestinal microbiota by prebiotics determines their fermentation activity, simultaneously influencing the SCFA level, which confers a health benefit on the host [139,140]. Moreover, prebiotics cause a reduction of intestinal pH and maintain the osmotic retention of water in the bowel [134]. However, it should be considered that an overdose of prebiotics may lead to flatulence and diarrhoea—these effects are absent in the case of excessive consumption of probiotics. Prebiotics may be consumed on a long-term basis and for prophylactic purposes. Moreover, when used at correct doses, they do not stimulate any adverse effects, such as diarrhoea, susceptibility to UV light, or hepatic injuries caused by antibiotics. Prebiotic substances are not allergenic and do not proliferate the abundance of antibiotic-resistance genes. Of course, the effect of the elimination of selected pathogens achieved by the use of prebiotics may be inferior to antibiotics, but the properties mentioned above make them a natural substitute for antibiotics [134].

### 3.2. Prebiotic Substances

The majority of identified prebiotics are carbohydrates of various molecular structures, naturally occurring in human and animal diets. The physiological properties of potential prebiotics determine their beneficial effect on the host’s health. Prebiotics may be classified according to those properties as [134]:

- not digested (or only partially digested);
- not absorbed in the small intestine;
- poorly fermented by bacteria in the oral cavity;
- well fermented by seemingly beneficial intestinal bacteria;
- poorly fermented by potential pathogens in the bowel.

Carbohydrates, such as dietary fibre, are potential prebiotics. Prebiotic and dietary fibre are terms used alternatively for food components that are not digested in the gastrointestinal tract. A significant difference between those two terms is that prebiotics are fermented by strictly defined groups of microorganisms, and dietary fibre is used by the majority of colonic microorganisms [141]. Therefore, considering one of the basic classification criteria, it turns out that using those terms alternatively is not always correct. Prebiotics may be a dietary fibre, but dietary fibre is not always a prebiotic [138]. The following non-starch polysaccharides are considered to be dietary fibre: cellulose, hemicellulose, pectins, gums, substances obtained from marine algae, as well as lactulose, soy oligosaccharides, inulins, fructooligosaccharides, galactooligosaccharides, xylooligosaccharides, and isomaltooligosaccharides. Based on the number of monomers bound together, prebiotics may be classified as: disaccharides, oligosaccharides (3–10 monomers), and polysaccharides. The most promising and fulfilling criteria for the classification of prebiotic substances, as evidenced by in vitro and in vivo studies, are oligosaccharides, including [142,143]: fructooligosaccharides (FOS), galactooligosaccharides (GOS), isomaltooligosaccharides (IMO), xylooligosaccharides (XOS), transgalactooligosaccharides (TOS), and soybean oligosaccharides (SBOS).

Also, polysaccharides such as inulin, reflux starch, cellulose, hemicellulose, or pectin may potentially be prebiotics. Examples of prebiotics that are most commonly used in human nutrition are presented in Table 4. The use of glucooligosaccharides, glicooligosaccharides, lactitol, izomaltooligosaccharides, stachyose, raffinose, and saccharose as prebiotics requires further studies [144].

### 3.3. Mechanism of Action of Prebiotics

Prebiotics are present in natural products, but they may also be added to food. The purpose of these additions is to improve their nutritional and health value. Some examples are: inulin, fructooligosaccharides, lactulose, and derivatives of galactose and *β*-glucans. Those substances may serve as a medium for probiotics. They stimulate their growth, and contain no microorganisms.

Figure 2 presents the principal mechanisms of prebiotic action and some of their effects on the host’s health. Prebiotics are not digested by host enzymes and reach the colon in a practically unaltered form, where they are fermented by saccharolytic bacteria (e.g., *Bifidobacterium* genus). The consumption of prebiotics largely affects the composition of the intestinal microbiota and its metabolic activity [147]. This is due to the modulation of lipid metabolism, enhanced absorbability of calcium, effect on the immunological system, and modification of the bowel function [147]. It is highly probable that providing an energy source that only specific species in the microbiota can utilize has a greater impact on microbiota composition and metabolism than these other factors. The molecular structure of prebiotics determines their physiological effects and the types of microorganisms that are able to use them as a source of carbon and energy in the bowel [134]. It was demonstrated that, despite the variety of carbohydrates that exhibit the prebiotic activity, the effect of their administration is an increased count of beneficial bacteria, mostly of the *Bifidobacterium* genus [148,149].

The mechanism of a beneficial effect of prebiotics on immunological functions remains unclear. Several possible models have been proposed [150]:

(1) Prebiotics are able to regulate the action of hepatic lipogenic enzymes by influencing the increased production of short-chain fatty acids (SCFAs), such as propionic acid.
(2) The production of SCFAs (especially of butyric acid) as a result of fermentation was identified as a modulator of histone acetylation, thus increasing the availability of numerous genes for transcription factors.
(3) The modulation of mucin production.
(4) It was demonstrated that FOS and several other prebiotics cause an increased count of lymphocytes and/or leukocytes in gut-associated lymphoid tissues (GALTs) and in peripheral blood.
(5) The increased secretion of IgA by GALTs may stimulate the phagocytic function of intra-inflammatory macrophages.

The main aim of prebiotics is to stimulate the growth and activity of beneficial bacteria in the gastrointestinal tract, which confers a health benefit on the host. Through mechanisms including antagonism (the production of antimicrobial substances) and competition for epithelial adhesion and for nutrients, the intestinal microbiota acts as a barrier for pathogens. Final products of carbohydrate metabolism are mostly SCFAs, namely: acetic acid, butyric acid, and propionic acid, which are subsequently used by the host as a source of energy [151]. As a result of the fermentation of carbohydrates, *Bifidobacterium* or *Lactobacillus* may produce some compounds inhibiting the development of gastrointestinal pathogens, as well as cause a reduction in the intestinal pH [152]. Moreover, *Bifidobacterium* genus bacteria demonstrate tolerance to the produced SCFAs and reduced pH. Therefore, due to their favourable effect on the development of beneficial intestinal bacteria, the administration of prebiotics may participate in the inhibition of the development of pathogens. There are very few documented study results regarding the inhibition of the development of pathogens by prebiotics. In 1997 and 2003, Bovee-Oudenhoven et al. studied the use of lactulose in the prevention of *Salmonella* Enteritidis infections on a rat model. Their results indicated that the acidification of the intestine occurring as a result of lactulose fermentation caused the reduced development of pathogens and increased translocation of pathogens from the bowel [153]. It was also demonstrated that the administration of prebiotics increases the absorption of minerals, mostly of magnesium and calcium [154,155].

### 3.4. Prebiotics for Humans

The presence of prebiotics in the diet may lead to numerous health benefits. Studies on colorectal carcinoma demonstrated that the disease occurs less commonly in people who often eat vegetables and fruit. This effect is attributed mostly to inulin and oligofructose [156]. Among the advantages of those prebiotics, one may also mention the reduction of the blood LDL (low-density lipoprotein) level, stimulation of the immunological system, increased absorbability of calcium, maintenance of correct intestinal pH value, low caloric value, and alleviation of symptoms of peptic ulcers and vaginal mycosis [157]. Other effects of inulin and oligofructose on human health are: the prevention of carcinogenesis, as well as the support of lactose intolerance or dental caries treatment [131]. Rat studies demonstrated that administration of inulin for five weeks caused a significant reduction of blood triacylglycerol levels [156]. Human studies demonstrated that the daily use of 12 g of inulin for one month led to the reduction of blood VLDL (very low-density lipoprotein) levels (the reduction of triacylglycerols by 27%, and of cholesterol by 5%). This effect is associated with the effect of the prebiotic on hepatic metabolism and the inhibition of acetyl-CoA carboxylase and of glukose-6-phosphate dehydrogenase. It is also supposed that oligofructose accelerates lipid catabolism [157].

Asahara et al. (2001) demonstrated a protective effect of galactooligosaccharides (GOS) in the prevention of *Salmonella* Typhimurium infections in a murine model [158]. Buddington et al. (2002) confirmed a positive effect of fructooligosaccharides (FOS) on protection against *Salmonella* Typhimurium and *Listeria monocytogenes* infections [159]. Moreover, prebiotics are helpful in combating pathogenic microorganisms, such as *Salmonella* Enteritidis and *Escherichia coli*, and reduce odour compounds [160]. There are many reports regarding the positive effect of prebiotics on the carcinogenesis process. Results of rat studies proved that a prebiotic-enriched diet leads to significantly reduced indexes of carcinogenesis. Scientific research demonstrated that butyric acid may be a chemopreventive factor in carcinogenesis [161], or an agent protecting against the development of colorectal carcinoma through the promotion of cell differentiation [162]. Besides butyric acid, propionic acid also may possess anti-inflammatory properties in relation to colorectal carcinoma cells. In vitro studies on human L97 and HT29 cell lines (representing early and late stages of colorectal carcinoma) demonstrated that inulin fractions in plasma supernatant caused a significant inhibition of growth and induction of apoptosis in human colorectal carcinoma [163]. According to scientific reports, the administration of inulin and oligofructose to rats caused the inhibition of azoxymethane-induced colorectal carcinoma at the growth stage [164]. The supplementation of inulin and oligofructose at the dose of 5%–15% had also an effect on reduced occurrence of breast cancer in rats and of metastases to lungs [165]. However, those results have to be confirmed in humans. Table 5 lists the results of studies focusing on the effect of prebiotics on human health. There are examples of clinical trials during which the prebiotics group received the prebiotic prophylactically or in addition to the standard therapy.

## 4. Synbiotics

Synbiotics are used not only for the improved survival of beneficial microorganisms added to food or feed, but also for the stimulation of the proliferation of specific native bacterial strains present in the gastrointestinal tract [179]. The effect of synbiotics on metabolic health remains unclear. It should be mentioned that the health effect of synbiotics is probably associated with the individual combination of a probiotic and prebiotic [180]. Considering a huge number of possible combinations, the application of synbiotics for the modulation of intestinal microbiota in humans seems promising [181].

### 4.1. Synbiotic Selection Criteria

The first aspect to be taken into account when composing a synbiotic formula should be a selection of an appropriate probiotic and prebiotic, exerting a positive effect on the host’s health when used separately. The determination of specific properties to be possessed by a prebiotic to have a favourable effect on the probiotic seems to be the most appropriate approach. A prebiotic should selectively stimulate the growth of microorganisms, having a beneficial effect on health, with simultaneous absent (or limited) stimulation of other microorganisms.

### 4.2. Synbiotics in Use

Previous sections discussed probiotic microorganisms and prebiotic substances most commonly used in human nutrition. A combination of *Bifidobacterium* or *Lactobacillus* genus bacteria with fructooligosaccharides in synbiotic products seems to be the most popular. Table 4 presents the most commonly used combinations of probiotics and prebiotics.

### 4.3. Mechanism of Action of Synbiotics

Considering the fact that a probiotic is essentially active in the small and large intestine, and the effect of a prebiotic is observed mainly in the large intestine, the combination of the two may have a synergistic effect [182]. Prebiotics are used mostly as a selective medium for the growth of a probiotic strain, fermentation, and intestinal passage. There are indications in the literature that, due to the use of prebiotics, probiotic microorganisms acquire higher tolerance to environmental conditions, including: oxygenation, pH, and temperature in the intestine of a particular organism [183]. However, the mechanism of action of an extra energy source that provides higher tolerance to these factors is not sufficiently explained. That combination of components leads to the creation of viable microbiological dietary supplements, and ensuring an appropriate environment allows a positive impact on the host’s health. Two modes of synbiotic action are known [184]:

(1) Action through the improved viability of probiotic microorganisms;
(2) Action through the provision of specific health effects.

The stimulation of probiotics with prebiotics results in the modulation of the metabolic activity in the intestine with the maintenance of the intestinal biostructure, development of beneficial microbiota, and inhibition of potential pathogens present in the gastrointestinal tract [180]. Synbiotics result in reduced concentrations of undesirable metabolites, as well as the inactivation of nitrosamines and cancerogenic substances. Their use leads to a significant increase of levels of short-chain fatty acids, ketones, carbon disulphides, and methyl acetates, which potentially results in a positive effect on the host’s health [184]. As for their therapeutic efficacy, the desirable properties of synbiotics include antibacterial, anticancerogenic, and anti-allergic effects. They also counteract decay processes in the intestine and prevent constipation and diarrhoea. It turns out that synbiotics may be highly efficient in the prevention of osteoporosis, reduction of blood fat and sugar levels, regulation of the immunological system, and treatment of brain disorders associated with abnormal hepatic function [185]. The concept of mechanisms of synbiotic action, based on the modification of intestinal microbiota with probiotic microorganisms and appropriately selected prebiotics as their substrates, is presented in Figure 1.

### 4.4. Synbiotics for Humans

Synbiotics have the following beneficial effects on humans [186]:

(1) Increased *Lactobacillus* and *Bifidobacterium* genus count and maintenance of balance of the intestinal microbiota;
(2) Improved hepatic function in patients suffering from cirrhosis;
(3) Improved immunomodulative abilities;
(4) Prevention of bacterial translocation and reduced incidence of nosocomial infections in patients’ post-surgical procedures and similar interventions.

The translocation of bacterial metabolism products, such as lipopolysaccharides (LPSs), ethanol, and short-chain fatty acids (SFCAs), leads to their penetration of the liver. SCFAs also stimulate the synthesis and storage of hepatic triacylglycerols. Those processes may intensify the mechanisms of hepatic detoxication, which may result in hepatic storage of triacylglycerol (IHTG), and intensify steatosis of the organ. A randomised trial on the use of a synbiotic containing five probiotics (*Lactobacillus plantarum*, *Lactobacillus delbrueckii* spp. *bulgaricus*, *Lactobacillus acidophilus*, *Lactobacillus rhamnosus*, *Bifidobacterium bifidum*) and inulin as a prebiotic in adult subjects with NASH (non-alcoholic steatohepatisis) demonstrated a significant reduction of IHTG (intrahepatic triacylglycerol) within six months [187]. It is also known that LPSs induce proinflammatory cytokines, such as the tumour necrosis factor alpha (TNF-*α*), playing a crucial role in insulin resistance and inflammatory cell uptake in NAFLD (non-alcoholic fatty liver disease). In the study on the effect of the synbiotic product containing a blend of probiotics (*Lactobacillus casei*, *Lactobacillus rhamnosus*, *Streptococcus thermophilus*, *Bifidobacterium breve*, *Lactobacillus acidophilu*s, *Bifidobacterium longum*, *Lactobacillus bulgaricus*) and fructooligosccharides, 52 adults participated for 28 weeks. It was found that supplementation with the synbiotic resulted in the inhibition of NF-κB (nuclear factor κB) and reduced production of TNF-*α* (tumour necrosis factor α) [188].

In rat studies, an increased level of intestinal IgA was found, following the introduction of the synbiotic product containing *Lactobacillus rhamnosus* and *Bifidobacterium lactis*, and inulin and oligofructose as prebiotics to the diet. Synbiotics lead to reduced blood cholesterol levels and lower blood pressure [157]. Moreover, synbiotics are used in the treatment of hepatic conditions [189] and improve the absorption of calcium, magnesium, and phosphorus [190].

Danq et al. (2013), in a meta-analysis, evaluated published studies on pro/prebiotics for eczema prevention, investigating bacterial strain efficacy and changes to the allergy status of the children involved. This meta-analysis found that probiotics or synbiotics may reduce the incidence of eczema in infants aged <2 years. Systemic sensitization did not change following probiotic administration [191].

Studies carried out within the framework of the SYNCAN project funded by the European Union verified the anti-carcinogenic properties of synbiotics. The effect of fructooligosaccharides (SYN1) combined with two probiotic strains (*Lactobacillus rhamnosus* GG and *Bifidobacterium animalis* subsp. *lactis* Bb12) on the health of patients at risk of colorectal cancer was studied. As a result, a change of biomarkers (genotoxicity, labelling index, labelled cells/crypt, transepithelial resistance, necrosis, interleukin 2, interferon γ) indicating the development of the disease in cancer patients, and in patients post polyp excision, was observed [192]. It was concluded that the application of the studied synbiotic may reduce the risk of colorectal carcinoma. A lower level of DNA damage was also observed, as well as a lower colonocyte proliferation ratio [147]. Table 6 lists the results of studies focusing on the effect of synbiotics on human health. There are examples of clinical trials during which the synbiotics group received the synbiotic prophylactically or in addition to the standard therapy.

## 5. Summary

Probiotic organisms are crucial for the maintenance of balance of human intestinal microbiota. Numerous scientific reports confirm their positive effect in the host’s health. Probiotic microorganisms are attributed a high therapeutic potential in, e.g., obesity, insulin resistance syndrome, type 2 diabetes, and non-alcohol hepatic steatosis [207]. It seems also that probiotics may be helpful in the treatment of irritable bowel syndrome, enteritis, bacterial infections, and various gastrointestinal disorders and diarrhoeas. Probiotic microorganisms are also effective in the alleviation of lactose intolerance and the treatment of atopic dermatitis. A positive effect of probiotics in the course of various neoplastic diseases and side effects associated with anti-cancer therapies is also worth noting. Prebiotics may be used as an alternative to probiotics, or as an additional support for them. It turns out that the development of bio-therapeutic formulas containing both appropriate microbial strains and synergistic prebiotics may lead to the enhancement of the probiotic effect in the small intestine and the colon. Those “enhanced” probiotic products may be even more effective, and their protective and stimulatory effect superior to their components administered separately [208]. It seems that we will see further studies on combinations of probiotics and prebiotics, and further development of synbiotics. Future studies may explain the mechanisms of actions of those components, which may confer a beneficial effect on human health.

## Figures and Tables

**Table 1:** Selection criteria of probiotic strains [5,20].

Criterion	Required Properties
**Safety**	Human or animal origin. Isolated from the gastrointestinal tract of healthy individuals. History of safe use. Precise diagnostic identification (phenotype and genotype traits). Absence of data regarding an association with infective disease. Absence of the ability to cleave bile acid salts. No adverse effects. Absence of genes responsible for antibiotic resistance localised in non-stable elements.
**Functionality**	Competitiveness with respect to the microbiota inhabiting the intestinal ecosystem. Ability to survive and maintain the metabolic activity, and to grow in the target site. Resistance to bile salts and enzymes. Resistance to low pH in the stomach. Competitiveness with respect to microbial species inhabiting the intestinal ecosystem (including closely related species). Antagonistic activity towards pathogens (e.g., *H. pylori*, *Salmonella* sp., *Listeria monocytogenes*, *Clostridium difficile*). Resistance to bacteriocins and acids produced by the endogenic intestinal microbiota. Adherence and ability to colonise some particular sites within the host organism, and an appropriate survival rate in the gastrointestinal system.
**Technological usability**	Easy production of high biomass amounts and high productivity of cultures. Viability and stability of the desired properties of probiotic bacteria during the fixing process (freezing, freeze-drying), preparation, and distribution of probiotic products. High storage survival rate in finished products (in aerobic and micro-aerophilic conditions). Guarantee of desired sensory properties of finished products (in the case of the food industry). Genetic stability. Resistance to bacteriophages.

**Table 2:** Probiotic microorganisms used in human nutrition [24,25,26].

Type *Lactobacillus*	Type *Bifidobacterium*	Other Lactic Acid Bacteria	Other Microorganisms
*L. acidophilus* ^(a),^* L. amylovorus ^(b),^* L. casei ^(a),(b),^* L. gasseri ^(a),^* L. helveticus ^(a),^* L. johnsonii ^(b),^* L. pentosus ^(b),^* L. plantarum ^(b),^* L. reuteri ^(a),^* L. rhamnosus ^(a),(b),^*	*B. adolescentis* ^(a)^ *B. animalis* ^(a),^* B. bifidum ^(a)^ *B. breve* ^(b)^ *B. infantis* ^(a)^ *B. longum* ^(a),^*	*Enterococcus faecium* ^(a)^ *Lactococcus lactis* ^(b),^* Streptococcus thermophilus ^(a),^*	*Bacillus clausii* ^(a),^* Escherichia coli Nissle 1917 ^(a)^ *Saccharomyces cerevisiae (boulardi)* ^(a),^*

^(a)^ Mostly as pharmaceutical products; ^(b)^ mostly as food additives; * QPS (Qualified Presumption of Safety) microorganisms.

**Table 3:** Examples of clinical trials regarding the effect of probiotics on human health.

References	Subjects	Microorganism	Time of Administration	Main Outcome
**Obesity**				
[90]	50 obese adolescents	*L. salivarius* Ls-33	12 weeks	Increase in the ratios of *Bacteroides*, *Prevotellae,* and *Porphyromonas*.
[91]	50 adolescents with obesity	*L. salivarius* Ls-33	12 weeks	No effect.
[92]	87 subjects with high BMI	*L. gasseri* SBT2055	12 weeks	Reduction in BMI, waist, abdominal VFA, and hip circumference.
[93]	210 adults with large VFA	*L. gasseri* SBT2055	12 weeks	Reduction in BMI and arterial BP values.
[94]	40 adults with obesity	*L. plantarum*	3 weeks	Reduction in BMI and arterial BP values.
[95,96,97]	75 subjects with high BMI	*L. acidophilus* La5, *B. lactis* Bb12, *L. casei* DN001	8 weeks	Changes in gene expression in PBMCs as well as BMI, fat percentage, and leptin levels.
[98]	70 overweight and obese subjects	*E. faecium* and 2, *S. thermophilus strains*	8 weeks	Reduction in body weight, systolic BP, LDL-C, and increase in fibrinogen levels.
[99]	60 overweight subjects	*Bifidobacterium*, *Lactobacillus*, *S. thermophilus*	6 weeks	Improvement in lipid profile, insulin sensitivity, and decrease in CRP.
[100]	58 obese PM women	*L. paracasei* N19	6 weeks	No effect.
[101]	156 overweight adults	*L. acidophilus* La5, *B. animalis* subsp. *lactis* Bb12	6 weeks	Reduction in fasting glucose concentration and increase in HOMA-IR.
**Insulin resistance syndrome**				
[102]	28 patients with IRS	*L. casei* Shirota	12 weeks	No effect.
[103]	30 patients with IRS	*L. casei* Shirota	12 weeks	Significant reduction in the VCAM-1 level.
[104]	24 PM women with IRS	*L. plantarum*	12 weeks	Glucose and homocysteine levels were significantly reduced.
**Type 2 diabetes**				
[105]	40 patients with T2D	*L. planatarum* A7	8 weeks	Decreased methylation process, SOD, and 8-OHDG.
[106]	45 patients with T2D	*L. acidophilus* La-5, *B. animalis* subsp. *lactis* BB-12	6 weeks	Significant difference between groups concerning mean changes of HbA1c, TC, and LDL-C.
[107]	44 patients with T2D	*L. acidophilus* La-5, *B. animalis* subsp. *lactis* BB-12	8 weeks	Increased HDL-C levels and decreased LDL-C/HDL-C ratio.
[108]	64 patients with T2D	*L. acidophilus* La5, *B. lactis* Bb12	6 weeks	Reduced fasting blood glucose and antioxidant status.
[109]	60 patients with T2D	*L. acidophilus* La5, *B. lactis* Bb12	6 weeks	TC and LDL-C improvement.
[110]	45 males with T2D	*L. acidophilus* NCFM	4 weeks	No effect.
**Non-alcoholic fatty liver disease**				
[111]	20 obese children with NAFLD	*L. rhamnosus* GG	8 weeks	Decreased ALT and PG-PS IgAg antibodies.
[112]	28 adult individuals with NAFLD	*L. bulgaris*, *S. thermophilus*	12 weeks	Decreased ALT and γ-GTP levels.
[113]	72 patients with NAFLD	*L.acidophilus* La5, *B. breve* subsp. *lactis* Bb12	8 weeks	Reduced serum levels of ALT, ASP, TC, and LDL-C.
[114]	44 obese children with NAFLD	*Bifidobacterium*, *Lactobacillus*, *S. thermophilus*	16 weeks	Improved fatty liver severity, decreased BMI, and increased GLP1/aGLP1.
**Irritable bowel syndrome (IBS), gastrointestinal disorders, elimination of *Helicobacter*, inflammatory bowel disease (IBD), diarrhoeas**				
[115]	59 adults infected with *H. pylori*	*L. acidophilus* La5, *B. lactis* Bb12	6 weeks	Inhibitory effect against *Helicobacter pylori*.
[116]	16 patients infected with *H. pylori*	*L. casei* Shirota	6 weeks	Inhibited growth of *Helicobacter pylori* (by 64% in the probiotic group, and by 33% in the control).
[117]	269 children with otitis media and/or respiratory tract infections	*S. cerevisiae* (*boulardii*)	No data	Diarrhoea was less common in children receiving probiotic yeast (7.5%) compared to those receiving placebo (23%). No negative side effects were observed.
[118]	77 patients with ulcerative colitis	Probiotic VSL#3	12 weeks	Remission in 42.9% of patients in the probiotic group, and in 15.7% of patients in the placebo group.
[119]	90 breastfed neonates with intestinal colic	*L. reuteri* ATCC 55730	6 months	Elimination of pain and symptoms associated with intestinal colic already after one week of the use of the probiotic.
**Atopic dermatitis**				
[120]	512 pregnant women and 474 their newborn infants	*L. rhamnosus* HN001	women—from 35 weeks gestation until 6 months if breastfeeding, infants—from birth to 2 years	Substantially reduced the cumulative prevalence of eczema in infants.
[121]	53 children with moderate of severe atopic dermatitis	*L. fermentum* VRI 033 PCC^TM^	8 weeks	Reduction in SCORAD.
[122]	156 mothers of high-risk children (i.e., positive family history of allergic disease) and their offspring	*B. bifidum*, *B. lactis*, *L. lactis*	Mothers—the last 6 weeks of pregnancy, offspring—12 months	Significantly reduction eczema in high-risk for a minimum of 2 years provided that the probiotic was administered to the infant within 3 months of birth.
[123]	50 children with AD	*B. animalis* subsp *lactis*	8 weeks	Significant reduction in the severity of AD with an improved ration of IFN-γ and IL-10.
**Alleviation of lactose intolerance**				
[124]	15 healthy, free-living adults with lactose maldigestion	*S. lactis*, *L. plantarum*, *S. cremoris*, *L. casei*, *S. diacetylactis*, *S. florentinus*, *L. cremoris*	1 day	Improved lactose digestion and tolerance.
[125]	44 patients	*B. animalis* subsp. *animal*is IM386 (DSM 26137), *L. plantarum* MP2026 (DSM 26329)	6 weeks	A significant lowering effect on diarrhoea and flatulence.
**Different types of cancer and side effects associated with cancer**				
[126]	100 patients with colorectal carcinoma	*L. plantarum* CGMMCC No 1258, *L. acidophilus* LA-11, *B. longum* BL-88	16 days	Improvement in the integrity of gut mucosal barrier and decrease in infections complications.
[127]	63 patients with diarrhoea during radiotherapy in cervical cancer	*L. acidophilus*, *B. bifidum*	7 weeks	Reduction in incidence of diarrhoea and better stool consistency.
[128]	150 patients diagnosed with colorectal cancer	*L. rhamnosus* 573	24 weeks	Patients had less grade 4 or 4 diarrhoea, less abdominal discomfort, needed less hospital care, and had fewer chemo dose reductions due to bowel toxicity.

Abbreviations: AD—atopic dermatitis; ALT—alanine amino transferase; ASP—aspartate amino transferase; BMI—body mass index; BP—blood pressure; CRP—C-reactive protein; γ-GTP—γ-glutamyltranspeptidase; GLP1—glucagon-like peptide 1; HDL-C—high-density lipoprotein cholesterol; HOMA-IR—homeostasis model assessment of insulin resistance; IL-10—interleukin 10; LDL-C—low-density lipoprotein cholesterol; NAFLD—non-alcoholic fatty liver disease; PBMC—peripheral blood mononuclear cell; PM—postmenopausal; SCORAD—SCORing Atopic Dermatitis; SOD—superoxide dismutase, sVCAM-1—soluble vascular cell adhesion molecule-1; TC—total cholesterol; T2D—type 2 diabetes; VFA—visceral fat area; 8-OHDG—8-hydroxy-2′-deoxyguanosine.

**Table 4:** Examples of prebiotics and synbiotics used in human nutrition [134,145,146].

Human Nutrition	
Prebiotics	Synbiotics
FOS GOS Inulin XOS Lactitol Lactosucrose Lactulose Soy oligosaccharides TOS	*Lactobacillus* genus bacteria + inulin *Lactobacillus*, *Streptococcus* and *Bifidobacterium* genus bacteria + FOS *Lactobacillus*, *Bifidobacterium*, *Enterococcus* genus bacteria + FOS *Lactobacillu*s and *Bifidobacterium* genus bacteria + oligofructose *Lactobacillu*s and *Bifidobacterium* genus bacteria + inulin

Abbreviations: FOS—fructooligosaccharides; GOS—galactooligosaccharides; TOS—transgalactooligosaccharides; XOS—xylooligosaccharides.

**Table 5:** Examples of clinical trials regarding the effect of prebiotics on human health.

References	Subjects	Prebiotic	Time of Administration	Main Outcome
**Obesity**				
[166]	48 healthy adults with a body mass index (in kg/m^2^) >25	OFS	12 weeks	There was a reduction in body weight of 1.03 ± 0.43 kg with oligofructose supplementation, whereas the control group experienced an increase in body weight of 0.45 ± 0.31 kg over 12 weeks (*p* = 0.01). Glucose decreased in the oligofructose group and increased in the control group between the initial and final tests (*p* ≤ 0.05). Insulin concentrations mirrored this pattern (*p* ≤ 0.05). Oligofructose supplementation did not affect plasma active glucagon-like peptide 1 secretion. According to a visual analogue scale designed to assess side effects, oligofructose was well tolerated.
**Insulin resistance syndrome**				
[167]	10 patients with type 2 diabetes	FOS	4 weeks (double repetition)	The plasma glucose response to a fixed exogenous insulin bolus did not differ at the end of the two periods. FOS had no effect on glucose and lipid metabolism in type 2 diabetics.
**Type 2 diabetes**				
[168]	15 subjects with type 2 diabetes	AX	5 weeks (double repetition)	A supplement of 15 g/day of AX-rich fibre can significantly improve glycaemic control in people with type 2 diabetes.
[169]	11 patients with impaired glucose tolerance	AX	6 weeks	No effects of arabinoxylan were observed for insulin, adiponectin, leptin, or resistin as well as for apolipoprotein B, and unesterified fatty acids. In conclusion, the consumption of AX in subjects with impaired glucose tolerance improved fasting serum glucose and triglycerides. However, this beneficial effect was not accompanied by changes in fasting adipokine concentrations.
**Non-alcoholic fatty liver disease**				
[170]	7 patients with non-alcoholic steatohepatitis	OFS	8 weeks	Compared to placebo, OFS significantly decreased serum aminotransferases, aspartate aminotransferase after 8 weeks, and insulin level after 4 weeks, but this could not be related to a significant effect on plasma lipids.
**Irritable bowel syndrome (IBS), gastrointestinal disorders, elimination of *Helicobacter*, inflammatory bowel disease (IBD), diarrhoeas**				
[171]	281 healthy infants (15 to 120 days)	GOS, FOS	12 months	Fewer episodes of acute diarrhoea, fewer upper respiratory tract infections.
[172]	160 healthy bottle-fed infants within 0–14 days after birth	GOS, FOS	3 months	Prebiotic formula well tolerated, normal growth trend toward a higher percentage of *Bifidobacterium* and a lower percentage of *E. coli* in stool, suppresses *Clostridium* in stool.
[173]	215 healthy infants	GOS, FOS	27 weeks	The concentration of secretory IgA was higher in the prebiotic group than the control; also, *Bifidobacterium* percentage was higher than the control and *Clostridium* was lower.
[174]	24 patients with chronic pouchitis	inulin	3 weeks	Inulin treatment resulted in decreased endoscopic and histological inflammation. This effect was associated with increased intestinal butyrate, lowered pH, and significantly decreased numbers of *Bacteroides fragilis*.
[175]	10 Crohn’s disease patients	FOS	3 weeks	Reduced disease activity index.
**Atopic dermatitis**				
[176]	259 infants at risk for atopy	GOS, FOS	6 months	Significant reduction of frequency of AD.
[177]	259 healthy term infants with a parental history of atopy	GOS, FOS	6 months	Prebiotic group had significantly lower allergic symptoms—AD, wheezing, urticaria, and fewer upper respiratory infections than controls during the first 2 years.
**Alleviation of lactose intolerance**				
[178]	85 lactose intolerant participants	GOS	36 days	71% of subjects reported improvements in at least one symptom (pain, bloating, diarrhoea, cramping, or flatulence). Also on day 36, populations of bifidobacteria significantly increased by 90% in 27 of the 30 non-lactose tolerant participants who took GOS. Lactose fermenting *Bifidobacterium*, *Faecalibacterium*, and *Lactobacillus* were all significantly increased.
**Different types of cancer and side effects associated with cancer**				
[163]	Human L97 and HT29 cell lines (representing early and late stages of colorectal carcinoma)	inulin	No data	Growth inhibition and induction of apoptosis in human colorectal carcinoma.

Abbreviations: AD—atopic dermatitis; AX—arabinoxylan; FOS—fructooligosaccharides; GOS—galactooligosaccharides; IgA—immunoglobulin A; OFS—oligofructose.

**Table 6:** Examples of clinical trials regarding the effect of synbiotics on human health.

References	Subjects	Composition of Synbiotic	Time of Administration	Main Outcome
**Obesity**				
[193]	153 obese men and women	*L. rhamnosus* CGMCC1.3724, inulin	36 weeks	Weight loss and reduction in leptin. Increase in Lachnospiraceae.
[194]	70 children and adolescents with high BMI	*L. casei*, *L. rhamnosus*, *S. thermophilus*, *B. breve*, *L. acidophilus*, *B. longum*, *L. bulgaricus*, FOS	8 weeks	Decrease in BMI z-score and waist circumference.
[195]	77 obese children	*L. acidophilus*, *L. rhamnosus*, *B. bifidum*, *B. longum*, *E. faecium*, FOS	4 weeks	Changes in anthropometric measurements. Decrease in TC, LDL-C, and total oxidative stress serum levels.
**Insulin resistance syndrome**				
[196]	38 subjects with IRS	*L. casei*, *L. rhamnosus*, *S. thermophilus*, *B. breve*, *L. acidophilus*, *B. longum*, *L. bulgaricus*, FOS	28 weeks	The levels of fasting blood sugar and insulin resistance improved significantly.
**Type 2 diabetes**				
[197]	54 patients with T2D	*L. acidophilus*, *L. casei*, *L. rhamnosus*, *L. bulgaricus*, *B. breve*, *B. longum*, *S. thermophilus*, FOS	8 weeks	Increased HOMA-IR and TGL plasma level; reduced CRP in serum.
[198]	81 patients with T2D	*L. sporogenes*, inulin	8 weeks	Significant reduction in serum insulin levels, HOMA-IR, and homeostatic model assessment cell function.
[199]	78 patients with T2D	*L. sporogenes*, inulin	8 weeks	Decrease in serum lipid profile (TAG, TC/HDL-C) and a significant increase in serum HDL-C levels.
[200]	20 patients with T2D	*L. acidophilus*, *B. bifidum*, oligofructose	2 weeks	Increased HDL-C and reduced fasting glycaemia.
**Non-alcoholic fatty liver disease**				
[187]	20 individuals with NASH	*L. plantarum*, *L. delbrueckii* spp*. bulgaricus*, *L. acidophilus*, *L. rhamnosus*, *B. bifidum*, inulin	26 weeks	Decreased IHTG content.
[188]	52 adult individuals with NAFLD	*L. casei*, *L. rhamnosus*, *S. thermophilus*, *B. breve*, *L. acidophilus*, *B. longum*, *L. bulgaricus*, FOS	30 weeks	Inhibition of NF-κB and reduction of TNF-*α*.
**Irritable bowel syndrome (IBS), gastrointestinal disorders, elimination of *Helicobacter*, inflammatory bowel disease (IBD), diarrhoeas**				
[201]	76 patients with IBS	*L. acidophilus La-5^®^*, *B. animalis* ssp. *lactis* BB-12*^®^*, dietary fibres (Beneo)	4 weeks	On average, an 18% improvement in total IBS-QoL score was reported and significant improvements in bloating severity, satisfaction with bowel movements, and the severity of IBS symptoms’ interference with patients’ everyday life were observed. However, there were no statistically significant differences between the synbiotic group and the placebo group.
[202]	69 children aged 6–16 years who had biopsy proven *H. pylori* infection	*B. lactis* B94, inulin	14 days	From a total of 69 *H. pylori*-infected children (female/male = 36/33; mean ± SD = 11.2 ± 3.0 years), eradication was achieved in 20 out of 34 participants in the standard therapy group and 27/35 participants in the synbiotic group. There were no significant differences in eradication rates between the standard therapy and the synbiotic groups.
[203]	40 patients with UC	*B. longum*, psyllium	4 weeks	Patients with UC on synbiotic therapy experienced greater quality-of-life changes than patients on probiotic or prebiotic treatment.
**Atopic dermatitis**				
[204]	90 infants with AD	*B. breve* M-16V, GOS and FOS mixture (Immunofortis^®^)	12 weeks	This synbiotic mixture did not have a beneficial effect on AD severity in infants, although it did successfully modulate their intestinal microbiota.
[205]	40 infants and children aged 3 months to 6 years with AD	*L. casei*, *L. rhamnosus*, *S. thermophilus*, *B. breve*, *L. acidophilus*, *B. infantis*, *L. bulgaricus*, FOS	8 weeks	A mixture of seven probiotic strains and FOS may clinically improve the severity of AD in young children.
**Alleviation of lactose intolerance**				
[206]	20 females and males	*Lactobacillus*, *Bifidobacterium*, FOS	5 weeks	Consumption of the probiotic mixture improved the gastrointestinal performance associated with lactose load in subjects with LI. Symptoms were additionally reduced by the addition of prebiotics. The supplementation was safe and well tolerated, with no significant adverse effect observed.
**Different types of cancer and side effects associated with cancer**				
[192]	43 polypeptomized and 37 colon cancer patients	*L. rhamnosus* GG, *B. lactis* Bb12, inulin	12 weeks	Increased *L. rhamnosus* and *B. lactis* in faeces, reduction in *C. perfringens*, prevents increased secretion of IL-2 in polypectomized patients, increased production of interferon-γ in cancer patients.

Abbreviations: BMI—body mass index; CFU—colony-forming-unit; CRP—C-reactive protein; FOS—fructo-oligossacharides; IBS-QoL—quality of life with IBS; HDL-C—high-density lipoprotein cholesterol; HOMA-IR—homeostasis model assessment of insulin resistance; IHTG—intrahepatic triacylglycerol; IRS—insulin resistance syndrome; LDL-C—low-density lipoprotein cholesterol; LI—lactose intolerance; NAFLD—non-alcoholic fatty liver disease; NF-κB—nuclear factor κB; T2D—type 2 diabetes; TAG—triacylglycerols; TC—total cholesterol; TGL—total glutathione levels; TNF-*α*—tumour necrosis factor α; UC—ulcerative colitis.

## References

[1] Vergin F. (1954). Anti-und Probiotica. Hipokrates.

[2] Lilly D.M., Stillwell R.H. (1965). Probiotics: Growth promoting factors produced by microorganisms. Science.

[3] Fuller R. (1989). Probiotics in man and animals. J. Appl. Microbiol..

[4] Guarner F., Schaafsma G.J. (1998). Probiotics. Int. J. Food Microbiol..

[5] Food and Agriculture Organization (FAO) Guidelines for the Evaluation of Probiotics in Food.

[6] Hill C., Guarner F., Reid G., Gibson G.R., Merenstein D.J., Pot B., Morelli L., Canani R.B., Flint H.J., Salminen S. (2014). Expert consensus document: The International Scientific Association for Probiotics and Prebiotics consensus statement on the scope and appropriate use of the term probiotic. Nat. Rev. Gastroenterol. Hepatol..

[7] Gibson R.G., Roberfroid M.B. (1995). Dietary modulation of the human colonic microbiota: Introducing the concept of prebiotics. J. Nutr..

[8] Gibson G.R., Probert H.M., van Loo J., Rastall R.A., Roberfroid M. (2004). Dietary modulation of the human colonic microbiota: Updating the concept of the prebiotics. Nutr. Res. Rev..

[9] Food and Agriculture Organization (2007). FAO Technical Meeting on Prebiotics: Food Quality and Standards Service (AGNS), Food and Agriculture Organization of the United Nations (FAO).

[10] Skalkam M.L., Wiese M., Nielsen D.S., van Zanten G. (2016). In Vitro Screening and Evaluation of Synbiotics.

[11] Cencic A., Chingwaru W. (2010). The role of functional foods, nutraceuticals, and food supplements in intestinal health. Nutrients.

[12] Rioux K.P., Madsen K.L., Fedorak R.N. (2005). The role of enteric microflora in inflammatory bowel disease: Human and animal studies with probiotics and prebiotics. Gastroenterol. Clin. N. Am..

[13] Bengmark S. (2005). Bioecological control of the gastrointestinal tract: The role of flora and supplemented probiotics and synbiotics. Gastroenterol. Clin. N. Am..

[14] Panesar P.S., Kaur G., Panesar R., Bera M.B. (2009). Synbiotics: Potential Dietary Supplements in Functional Foods.

[15] Hosono A., Nakazawa Y., Hosono A. (1992). Fermented milk in the orient. Functions of Fermented Milk: Challengers for the Health Sciences.

[16] Miecznikow E. (1907). O naturze ludzkiej—Zarys Filozofii Optymistycznej.

[17] Lee Y.K., Lee Y.K., Salminen S. (2009). Selection and maintenance of probiotic microorganisms. Handbook of Probiotics and Prebiotics.

[18] Sanders M.E., Gibson G., Harsharnjit S.G., Guarner F. (2007). Probiotics: Their Potential to Impact Human Health.

[19] Sanders M.E., Lenoir-Wijnkoop I., Salminen S., Merenstein D.J., Gibson G.R., Petschow B.W., Nieuwdorp M., Tancredi D.J., Cifelli C.J., Jacques P. (2014). Probiotics and prebiotics: Prospects for public health and nutritional recommendations. Ann. N. Y. Acad. Sci..

[20] European Food Safety Authority (EFSA) (2005). Opinion of the Scientific Committee on a request from EFSA related to a generic approach to the safety assessment by EFSA of microorganisms used in food/feed and the production of food/feed additives. EFSA J..

[21] Simon O. (2005). Micro-organisms as feed additives—Probiotics. Adv. Pork Prod..

[22] Anadón A., Martínez-Larrańaga M.R., Martínez M.A. (2006). Probiotics for animal nutrition in the European Union. Regulation and safety assessment. Regul. Toxicol. Pharmacol..

[23] Gaggia F., Mattarelli P., Biavati B. (2010). Probiotics and prebiotics in animal feeding for safe food production. Int. J. Food Microbiol..

[24] European Food Safety Authority (EFSA) (2013). The European Union Summary report on trends and sources of zoonoses, zoonotic agents and food-borne outbreaks in 2011. EFSA J..

[25] European Food Safety Authority (EFSA) (2013). Scientific opinion on the maintenance of the list of QPS biological agents intentionally added to food and feed (2013 update). EFSA J..

[26] European Food Safety Authority (EFSA) (2017). Scientific Opinion on the update of the list of QPS-recommended biological agents intentionally added to food or feed as notified to EFSA (2017 update). EFSA J..

[27] Schachtsiek M., Hammes W.P., Hertel C. (2004). Characterization of *Lactobacillus* coryniformis DSM 20001T surface protein CPF mediating coaggregation with and aggregation among pathogens. Appl. Environ. Microbiol..

[28] Oelschlaeger T.A. (2010). Mechanisms of probiotic actions—A review. Int. J. Med. Microbiol..

[29] Cremonini F., di Caro S., Nista E.C., Bartolozzi F., Capelli G., Gasbarrini G., Gasbarrini A. (2002). Meta-analysis: The eqect of probiotic administration on antibiotic associateddiarrhoea. Aliment. Pharmacol. Ther..

[30] Johnston B.C., Supina A.L., Vohra S. (2006). Probiotics for pediatric antibiotic-associated diarrhea: A meta-analysis of randomized placebo-controlled trials. Can. Med. Assoc. J..

[31] Schoster A., Kokotovic B., Permin A., Pedersen P.D., Dal Bello F., Guardabassi L. (2013). In Vitro inhibition of *Clostridium difficile* and *Clostridium perfringens* by commercial probiotic strains. Anaerobe.

[32] JimmySaint-Cyr M., Haddad N., Taminiau B., Poezevara T., Quesne S., Amelot M., Daube G., Chemaly M., Dousset X., Guyard-Nicodème M. (2017). Use of the potential probiotic strain *Lactobacillus salivarius* SMXD51 to control *Campylobacter jejuni* in broilers. Int. J. Food Microbiol..

[33] Carter A., Adams M., La Ragione R.M., WoodWard M.J. (2017). Colonisation of poultry by *Salmonella* Enteritidis S1400 is reduced by combined administration of *Lactobacillus salivarius* 59 and *Enterococcus faecium* PXN-33. Vet. Microbiol..

[34] Chingwaru W., Vidmar J. (2017). Potential of Zimbabwean commercial probiotic products and strains of *Lactobacillus plantarum* as prophylaxis and therapy against diarrhoea caused by *Escherichia coli* in children. Asian Pac. J. Trop. Med..

[35] Hussain S.A., Patil G.R., Reddi S., Yadav V., Pothuraju R., Singh R.R.B., Kapila S. (2017). *Aloe vera* (*Aloe barbadensis* Miller) supplemented probiotic *lassi* prevents *Shigella* infiltration from epithelial barrier into systemic blood flow in mice model. Microb. Pathog..

[36] Sikorska H., Smoragiewicz W. (2013). Role of probiotics in the prevention and treatment of ethicillin-resistant *Staphylococcus* aureus infections. Int. J. Antimicrob. Agents.

[37] De Montijo-Prieto S., Moreno E., Bergillos-Meca T., Lasserrot A., Ruiz-López M., Ruiz-Bravo A., Jimenez-Valera M. (2015). A *Lactobacillus plantarum* strain isolated from kefir protects against intestinal infection with *Yersinia enterocolitica* O9 and modulates immunity in mice. Res. Microbiol..

[38] Thomas D.W., Greer F. (2010). Probiotics and prebiotics in pediatrics. Pediatrics.

[39] Heczko P., Strus M., Jawień M., Szymański H. (2005). Medyczne zastosowanie probiotyków. Wiad. Lek..

[40] Kumar S., Bansal A., Chakrabarti A., Singhi S. (2013). Evaluation of efficacy of probiotics in prevention of *Candida* colonization in a PICU—A randomized controlled trial. Crit. Care Med..

[41] Nase L., Hatakka K., Savilahti E. (2001). Eqect of long-term consumption of *Lactobacillus* GG in milk on dental caries and caries risk in children. Caries Res..

[42] Li P., Gu Q. (2016). Complete genome sequence of *Lactobacillus plantarum* LZ95, a potential probiotic strain producing bacteriocins and B-group vitamin riboflavin. J. Biotechnol..

[43] Gu Q., Zhang C., Song D., Li P., Zhu X. (2015). Enhancing vitamin B12 content in soy-yogurt by *Lactobacillus reuteri*. Int. J. Food Microbiol..

[44] Pompei A., Cordisco L., Amaretti A., Zanoni S., Matteuzzi D., Rossi M. (2007). Folate production by bifidobacteria as a potential probiotic property. Appl. Environ. Microbiol..

[45] Nova E., Warnberg J., Gomez-Martinez S., Diaz L.E., Romeo J., Marcos A. (2007). Immunodulatory effects of probiotics in different stages of life. Br. J. Nutr..

[46] Mishra C., Lambert J. (1996). Production of anti-microbial substances by probiotics. Asia Pac. J. Clin. Nutr..

[47] Ouwehand A.C., Kirjavainen P.V., Shortt C., Salminen S. (1999). Probiotics: Mechanisms and established effects. Int. Dairy J..

[48] Reid G., McGroarty J.A., Angotti R., Cook R.L. (1988). *Lactobacillus* inhibitor production against *Escherichia coli* and coaggregation ability with uropathogens. Can. J. Microbiol..

[49] Schellenberg J., Smoragiewicz W., Karska-Wysocki B. (2006). A rapid method combining immunofluorescence and flow cytometry for improved understanding of competitive interactions between lactic acid bacteria (LAB) and methicillin-resistant *S. aureus* (MRSA) in mixed culture. J. Microbiol. Methods.

[50] Ishikawa H., Akedo I., Otani T., Suzuki T., Nakamura T., Takeyama I., Ishiguro S., Miyaoka E., Sobue T., Kakizoe T. (2005). Randomized trial of dietary fiber and *Lactobacillus casei* administration for prevention of colorectal tumors. Int. J. Cancer.

[51] Vandenbergh P.A. (1993). Lactic acid bacteria, their metabolic products and interference with microbial growth. FEMS Microbiol. Rev..

[52] Guillot J.F. (2003). Probiotic feed additives. J. Vet. Pharmacol. Ther..

[53] Isolauri E., Sutas Y., Kankaanpaa P., Arvilommi H., Salminen S. (2001). Probiotics: Effects on immunity. Am. J. Clin. Nutr..

[54] Brandao R.L., Castro I.M., Bambirra E.A., Amaral S.C., Fietto L.G., Tropia M.J.M. (1998). Intracellular signal triggered by cholera toxin in *Saccharomyces boulardii* and Saccharomyces cerevisiae. Applied Environ. Microbiol..

[55] Collado M.C., Meriluoto J., Salminen S. (2007). Interactions between pathogens and lactic acid bacteria: Aggregation and coaggregation abilities. Eur. J. Food Res. Technol..

[56] Begley M., Hill C., Gahan C.G.M. (2006). Bile salt hydrolase activity in probiotics. Appl. Environ. Microbiol..

[57] Weinberg E.D. (1997). The *Lactobacillus* anomaly: Total iron abstinence. Perspect. Biol. Med..

[58] Elli M., Zink R., Rytz A., Reniero R., Morelli L. (2000). Iron requirement of *Lactobacillus* spp. in completely chemically defined growth media. J. Appl. Microbiol..

[59] Borchers A.T., Selmi C., Meyers F.J., Keen C.L., Gershwin M.E. (2009). Probiotics and immunity. J. Gastroenterol..

[60] Gill H.S., Cross M.L., Calder P.C., Field C.J., Gill H.S. (2002). Probiotics and immune function. Nutrition and Immune Function.

[61] Marteau P., Shanahan F. (2003). Basic aspects and pharmacology of probiotics: An overview of pharmacokinetics, mechanisms of action and side-effects. Best Pract. Res. Clin. Gastroenterol..

[62] Schatzmayr G., Zehner F., Taubel M., Schatzmayr D., Klimitsch A., Loibner A.P., Binder E.M. (2006). Microbiologicals for deactivating mycotoxins. Mol. Nutr. Food Res..

[63] McCormick S.P. (2013). Microbial detoxification of mycotoxins. J. Chem. Ecol..

[64] Nikbakht Nasrabadi E., Jamaluddin R., Abdul Mutalib M.S., Khaza’ai H., Khalesi S., Mohd Redzwan S. (2013). Reduction of aflatoxin level in aflatoxin-induced rats by the activity of probiotic *Lactobacillus casei* strain Shirota. J. Appl. Microbiol..

[65] Upadrasta A., Madempudi R.S. (2016). Probiotics and blood pressure: Current insights. Integr. Blood Press. Control.

[66] Khalesi S., Sun J., Buys N., Jayasinghe R. (2014). Effect of probiotics on blood pressure. A Systematic review and meta-analysis of randomized, controlled trials. Hypertension.

[67] Ruan Y., Sun J., He J., Chen F., Chen R., Chen H. (2015). Effect of probiotics on glycemic control: A systematic review and meta-analysis of randomized, controlled trials. PLoS ONE.

[68] Lima-Filho J.V.M., Vieira E.C., Nicoli J.R. (2000). *Saccharomyces boulardii* and *Escherichia coli* combinations against experimential infections with *Shigella flexneri* and *Salmonella enteritidis* subsp. Typhimurium. J. Appl. Microbiol..

[69] Bengmark S. (2007). Bioecological control of inflammatory bowel disease. Clin. Nutr..

[70] Geier M.S., Butler R.N., Howarth G.S. (2007). Inflammatory bowel disease: Current insights into pathogenesis and new therapeutic options; probiotics, prebiotics and synbiotics. Int. J. Food Microbiol..

[71] Montalto M., Curigliano V., Santoro L., Vastola M., Cammarota G., Manna R., Gasbarrini A., Gasbarrini G. (2006). Management and treatment of lactose malabsorption. World J. Gastroenterol..

[72] Levri K.M., Ketvertis K., Deramo M., Merenstein J.H., D’Amico F. (2005). Do probiotics reduce adult lactose intolerance?. J. Family Pract..

[73] Geier M.S., Butler R.N., Howarth G.S. (2006). Probiotics, prebiotics and synbiotics: A role in chemoprevention for colorectal cancer?. Cancer Biol. Ther..

[74] Lesbros-Pantoflickova D., Corthesy-Theulaz I., Blum A.L. (2007). Helicobacter pylori and probiotics. J. Nutr..

[75] Guarner F., Khan A.G., Garisch J., Eliakim R., Gangl A., Thomson A., Krabshuis J., Lemair T. (2011). Probiotics and Prebiotics.

[76] Falagas M.E., Betsi G.I., Tokas T., Athanasiou S. (2006). Probiotics for prevention of recurrent urinary tract infections in women: A review of the evidence from microbiological and clinical studies. Drugs.

[77] Anukam K.C., Osazuwa E., Osemene G.I., Ehigiagbe F., Bruce A.W., Reid G. (2006). Clinical study comparing probiotic *Lactobacillus* GR-1 and RC-14 with metronidazole vaginal gel to treat symptomatic bacterial vaginosis. Microbes Infect..

[78] Reid G., Bruce A.W. (2005). Probiotics to prevent urinary tract infections: The rationale and evidence. World J. Urol..

[79] Kukkonen K., Savilahti E., Haahtela T., Juntunen-Backman K., Korpela R., Poussa T., Tuure T., Kuitunen M. (2007). Probiotics and prebiotic galacto-oligosaccharides in the prevention of allergic diseases: A randomized, double-blind, placebo-controlled trial. J. Allergy Clin. Immunol..

[80] Simons L.A., Amansec S.G., Conway P. (2006). Effect of *Lactobacillus* fermentum on serum lipids in subjects with elevated serum cholesterol. Nutr. Metab. Cardiovasc. Dis..

[81] Lieske J.C., Goldfarb D.S., de Simone C., Regnier C. (2005). Use of a probiotic to decrease enteric hyperoxaluria. Kidney Int..

[82] Rousseaux C., Thuru X., Gelot A., Barnich N., Neut C., Dubuquoy L., Dubuquoy C., Merour E., Geboes K., Chamaillard M. (2006). *Lactobacillus* acidophilus modulates intestinal pain and induces opioid and cannabinoid receptors. Nat. Med..

[83] Billoo A.G., Memon M.A., Khaskheli S.A., Murtaza G., Iqbal K., Saeed Shekhani M., Siddiqi A.Q. (2006). Role of a probiotic (*Saccharomyces boulardii*) in management and prevention of diarrhoea. World J. Gastroenterol..

[84] Parvez S., Malik K.A., Ah Kang S., Kim H.Y. (2006). Probiotics and their fermented food products are beneficial for health. J. Appl. Microbiol..

[85] McFarland L.V. (2006). Meta-Analysis of probiotics for the prevention of antibiotic associated diarrhea and the treatment of *Clostridium difficile* disease. Am. J. Gastroenterol..

[86] Hatakka K., Savilahti E., Pönkä A., Meurman J.H., Poussa T., Näse L., Saxelin M., Ko R. (2001). Effect of long term consumption of probiotic milk on infections in children attending day care centres: Double blind, randomised trial. Br. Med. J..

[87] Olivares M., Diaz-Ropero M., Gomez N., Sierra S., Lara-Villoslada F., Martin R., Miguel Rodriguez J., Xaus J. (2006). Dietary deprivation of fermented foods causes a fall in innate immune response. Lactic acid bacteria can counteract the immunological effect of this deprivation. J. Dairy Res..

[88] Olivares M., Diaz-Ropero M.A., Gomez N., Lara-Villoslada F., Sierra S., Maldonado J.A., Martin R., Lopez-Huertas E., Rodriguez J.M., Xaus J. (2006). Oral administration of two probiotic strains, *Lactobacillus* gasseri CECT5714 and *Lactobacillus* coryniformis CECT5711, enhances the intestinal function of healthy adults. Int. J. Food Microbiol..

[89] Alvaro E., Andrieux C., Rochet V., Rigottier-Gois L., Leprcq P., Sutren M., Galan P., Duval Y., Juste C., Dore J. (2007). Composition and metabolism of the intestinal microbiota in consumers and non-consumers of yogurt. Br. J. Nutr..

[90] Larsen N., Vogensen F.K., Gøbel R.J., Michaelsen K.F., Forssten S.D., Lahtinen S.J., Jakobsen M. (2013). Effect of *Lactobacillus* salivarius Ls-33 on fecal microbiota in obese adolescents. Clin. Nutr..

[91] Gøbel R.J., Larsen N., Jakobsen M., Mølgaard C., Michaelsen K.F. (2012). Probiotics to adolescents with obesity: Effects on inflammation and metabolic syndrome. J. Pediatr. Gastroenterol. Nutr..

[92] Kadooka Y., Sato M., Imaizumi K., Ogawa A., Ikuyama K., Akai Y., Okano M., Kagoshima M., Tsuchida T. (2010). Regulation of abdominal adiposity by probiotics (*Lactobacillus gasseri* SBT2055) in adults with obese tendencies in a randomized controlled trial. Eur. J. Clin. Nutr..

[93] Kadooka Y., Sato M., Ogawa A., Miyoshi M., Uenishi H., Ogawa H., Ikuyama K., Kagoshima M., Tsuchida T. (2013). Effect of *Lactobacillus* gasseri SBT2055 in fermented milk on abdominal adiposity in adults in a randomised controlled trial. Br. J. Nutr..

[94] Sharafedtinov K.K., Plotnikova O.A., Alexeeva R.I., Sentsova T.B., Songisepp E., Stsepetova J., Smidt I., Mikelsaar M. (2013). Hypocaloric diet supplemented with probiotic cheese improves body mass index and blood pressure indices of obese hypertensive patients—A randomized double-blind placebo-controlled pilot study. Nutr. J..

[95] Zarrati M., Salehi E., Mofid V., Hossein Zadeh-Attar M.J., Nourijelyani K., Bidad K., Shidfar F. (2013). Relationship between probiotic consumption and IL-10 and IL-17 secreted by PBMCs in overweight and obese people. Iran. J. Allergy Asthma Immunol..

[96] Zarrati M., Salehi E., Nourijelyani K., Mofid V., Zadeh M.J., Najafi F., Ghaflati Z., Bidad K., Chamari M., Karimi M. (2014). Effects of probiotic yogurt on fat distribution and gene expression of proinflammatory factors in peripheral blood mononuclear cells in overweight and obese people with or without weight-loss diet. J. Am. Coll. Nutr..

[97] Zarrati M., Shidfar F., Nourijelyani K., Mofid V., Hossein zadeh-Attar M.J., Bidad K., Najafi F., Gheflati Z., Chamari M., Salehi E. (2013). *Lactobacillus* acidophilus La5, *Bifidobacterium* BB12, and *Lactobacillus casei* DN001 modulate gene expression of subset specific transcription factors and cytokines in peripheral blood mononuclear cells of obese and overweight people. Biofactors.

[98] Agerholm-Larsen L., Raben A., Haulrik N., Hansen A.S., Manders M., Astrup A. (2000). Effect of 8 week intake of probiotic milk products on risk factors for cardiovascular diseases. Eur. J. Clin. Nutr..

[99] Rajkumar H., Mahmood N., Kumar M., Varikuti S.R., Challa H.R., Myakala S.P. (2014). Effect of probiotic (VSL#3) and W-3 on lipid profile, insulin sensitivity, inflammatory markers, and gut colonization in overweight adults: A randomized, controlled trial. Mediat. Inflamm..

[100] Brahe L.K., Le Chatelier E., Prifti E., Pons N., Kennedy S., Blædel T., Håkansson J., Dalsgaard T.K., Hansen T., Pedersen O. (2015). Dietary modulation of the gut microbiota—A randomised controlled trial in obese postmenopausal women. Br. J. Nutr..

[101] Ivey K.L., Hodgson J.M., Kerr D.A., Thompson P.L., Stojceski B., Prince R.L. (2015). The effect of yoghurt and its probiotics on blood pressure and serum lipid profile; a randomised controlled trial. Nutr. Metab. Cardiovasc. Dis..

[102] Leber B., Tripolt N.J., Blattl D., Eder M., Wascher T.C., Pieber T.R., Stauber R., Sourij H., Oettl K., Stadlbauer V. (2012). The influence of probiotic supplementation on gut permeability in patients with metabolic syndrome: An open label, randomized pilot study. Eur. J. Clin. Nutr..

[103] Tripolt N.J., Leber B., Blattl D., Eder M., Wonisch W., Scharnagl H., Stojakovic T., Obermayer-Pietsch B., Wascher T.C., Pieber T.R. (2013). Effect of supplementation with *Lactobacillus casei* Shirota on insulin sensitivity, β-cell function, and markers of endothelial function and inflammation in subjects with metabolic syndrome—A pilot study. J. Dairy Sci..

[104] Barreto F.M., Colado Simão A.N., Morimoto H.K., Batisti Lozovoy M.A., Dichi I., da Silva Miglioranza H.L. (2014). Beneficial effects of *Lactobacillus* plantarum on glycemia and homocysteine levels in postmenopausal women with metabolic syndrome. Nutrition.

[105] Hariri M., Salehi R., Feizi A., Mirlohi M., Ghiasvand R., Habibi N. (2015). A randomized, double-blind, placebo-controlled, clinical trial on probiotic soy milk and soy milk: Effects on epigenetics and oxidative stress in patients with type II diabetes. Genes Nutr..

[106] Tonucci L.B., Olbrich Dos Santos K.M., Licursi de Oliveira L., Rocha Ribeiro S.M., Duarte Martino H.S. (2015). Clinical application of probiotics in type 2 diabetes mellitus: A randomized, double-blind, placebo-controlled study. Clin. Nutr..

[107] Mohamadshahi M., Veissi M., Haidari F., Javid A.Z., Mohammadi F., Shirbeigi E. (2014). Effects of probiotic yogurt consumption on lipid profile in type 2 diabetic patients: A randomized controlled clinical trial. J. Res. Med. Sci..

[108] Ejtahed H.S., Mohtadi-Nia J., Homayouni-Rad A., Niafar M., Asghari-Jafarabadi M., Mofid V. (2012). Probiotic yogurt improves antioxidant status in type 2 diabetic patients. Nutrition.

[109] Ejtahed H.S., Mohtadi-Nia J., Homayouni-Rad A., Niafar M., Asghari-Jafarabadi M., Mofid V., Akbarian-Moghari A. (2011). Effect of probiotic yogurt containing *Lactobacillus acidophilus* and *Bifidobacterium lactis* on lipid profile in individuals with type 2 diabetes mellitus. J. Dairy Sci..

[110] Andreasen A.S., Larsen N., Pedersen-Skovsgaard T., Berg R.M., Møller K., Svendsen K.D., Jakobsen M., Pedersen B.K. (2010). Effects of *Lactobacillus* acidophilus NCFM on insulin sensitivity and the systemic inflammatory response in human subjects. Br. J. Nutr..

[111] Vajro P., Mandato C., Licenziati M.R., Franzese A., Vitale D.F., Lenta S., Caropreso M., Vallone G., Meli R. (2011). Effects of *Lactobacillus* rhamnosus strain GG in pediatric obesity-related liver disease. J. Gastroenterol. Nutr..

[112] Aller R., de Luis D.A., Izaola O., Conde R., Gonzalez Sagrado M., Primo D., de la Fuente B., Gonzalez J. (2011). Effect of a probiotic on liver aminotransferases in nonalcoholic fatty liver disease patients: A double blind randomized clinical trial. Eur. Rev. Med. Pharmacol. Sci..

[113] Nabavi S., Rafraf M., Somi M.H., Homayouni-Rad A., Asghari-Jafarabadi M. (2014). Effects of probiotic yogurt consumption on metabolic factors in individuals with nonalcoholic fatty liver disease. J. Dairy Sci..

[114] Alisi A., Bedogni G., Baviera G., Giorgio V., Porro E., Paris C., Giammaria P., Reali L., Anania F., Nobili V. (2014). Randomised clinical trial: The beneficial effects of VLS_3 in obese children with non-alcoholic steatohepatitis. Aliment. Pharmacol. Ther..

[115] Wang K.Y., Li S.N., Liu C.S., Perng D.S., Su Y.C., Wu D.C., Jan C.M., Lai C.H., Wang T.N., Wang W.M. (2005). Effects of ingesting *Lactobacillus*- and *Bifidobacterium*-containing yogurt in subjects with colonized Helicobacter pylori. Am. J. Clin. Nutr..

[116] Cats A., Kuipers E.J., Bosschaert M.A., Pot R.G., Vandenbroucke-Grauls C.M., Kusters J.G. (2003). Effect of frequent consumption of *Lactobacillus casei*—Containing milk drink in Helicobacter pylori-colonized subjects. Aliment. Pharmacol. Ther..

[117] Kotowska M., Albrecht P., Szajewska H. (2005). *Saccharomyces boulardii* in the prevention of antibiotic-associated diarrhoea in children: A randomized double-blind placebo-controlled trial. Aliment. Pharmacol. Ther..

[118] Sood A., Midha V., Makharia G.K., Ahuja V., Singal D., Goswami P., Tandon R.K. (2009). The probiotic preparation, VSL#3 induces remission in patients with mild-to-moderately active ulcerative colitis. Clin. Gastroenterol. Hepatol..

[119] Niv E., Naftali T., Hallak R., Vaisman N. (2005). The efficacy of *Lactobacillus reuteri* ATCC 55730 in the treatment of patients with irritable bowel syndrome–A double blind, placebo-controlled, randomized study. Clin. Nutr..

[120] Wickens K., Black P.N., Stanley T.V., Mitchell E., Fitzharris P., Tannock G.W., Purdie G., Crane J. (2008). A differential effect of 2 probiotics in the prevention of eczema and atopy: A double-blind, randomized, placebo-controlled trial. J. Allergy Clin. Immunol..

[121] Weston S., Halbert A., Richmond P., Prescott S.L. (2005). Effects of probiotics on atopic dermatitis: A randomised controlled trial. Arch. Dis. Child..

[122] Niers L., Martín R., Rijkers G., Sengers F., Timmerman H., van Uden N., Smidt H., Kimpen J., Hoekstra M. (2009). The effects of selected probiotic strains on the development of eczema (the PandA study). Allergy.

[123] Gøbel R.J., Larsen N., Mølgaard C., Jakobsen M., Michaelsen K.F. (2010). Probiotics to young children with atopic dermatitis: A randomized placebo-controlled trial. Int. J. Probiotics Prebiotics.

[124] Hertzler S.R., Clancy S.M. (2003). Kefir improves lactose digestion and tolerance in adults with lactose maldigestion. J. Am. Dietetic Assoc..

[125] Roškar I., Švigelj K., Štempelj M., Volfand J., Štabuc B., Malovrh Š., Rogelj I. (2017). Effects of a probiotic product containing *Bifidobacterium animalis* subsp. animalis IM386 and *Lactobacillus plantarum* MP2026 in lactose intolerant individuals: Randomized, placebo-controlled clinical trial. J. Funct. Foods.

[126] Liu Z., Qin H., Yang Z., Xia Y., Liu W., Yang J., Jiang Y., Zhang H., Wang Y., Zheng Q. (2011). Randomized clinical trial: The effects of perioperative probiotic treatment on barrier function and post-operative infectious complications in colorectal cancer surgery—A double-blind study. Aliment. Pharmacol. Ther..

[127] Chitapanarux I., Chitapanarux T., Traisathit P., Kudumpee S., Tharavichitkul E., Lorvidhaya V. (2010). Randomized controlled trial of live *Lactobacillus acidophilus* plus *Bifidobacterium bifidum* in prophylaxis of diarrhea during radiotherapy in cervical cancer patients. Radiat. Oncol..

[128] Österlund P., Ruotsalainen T., Korpela R., Saxelin M., Ollus A., Valta P., Kouri M., Elomaa I., Joensuu H. (2007). *Lactobacillus* supplementation for diarrhoea related to chemotherapy of colorectal cancer: A randomised study. Br. J. Cancer.

[129] Chung W.S.F., Walker A.W., Louis P., Parkhill J., Vermeiren J., Bosscher D., Duncan S.H., Flint H.J. (2016). Modulation of the human gut microbiota by dietary fibres occurs at the species level. BMC Biol..

[130] Crittenden R., Playne M.J., Lee Y.K., Salminen S. (2008). Nutrition News. Facts and functions of prebiotics, probiotics and synbiotics. Handbook of Probiotics and Prebiotics.

[131] Jakubczyk E., Kosikowska M. (2000). Nowa generacja mlecznych produktów fermentowanych z udziałem probiotyków i prebiotyków, produkty synbiotyczne. Prz. Mlecz..

[132] Wang Y. (2009). Prebiotics: Present and future in food science and technology. Food Res. Int..

[133] Maccfarlane G.T., Steed H., Maccfarlane S. (2008). Bacterial metabolism and health-related effects of galacto-oligosaccharides and other prebiotics. J. Appl. Microbiol..

[134] Crittenden R., Playne M.J., Lee Y.K., Salminen S. (2009). Prebiotics. Handbook of Probiotics and Prebiotics.

[135] Huebner J., Wehling R.L., Parkhurst A., Hutkins R.W. (2008). Effect of processing conditions on the prebiotic activity of commercial prebiotics. Int. Dairy J..

[136] Ze X., Duncan S.H., Louis P., Flint H.J. (2012). *Ruminococcus bromii* is a keystone species for the degradation of resistant starch in the human colon. ISME J..

[137] Angelakis E. (2017). Weight gain by gut microbiota manipulation in productive animals. Microb. Pathog..

[138] Śliżewska K., Nowak A., Barczyńska R., Libudzisz Z. (2013). Prebiotyki—Definicja, właściwości i zastosowanie w przemyśle. Żywność Nauka Technologia Jakość.

[139] Sivieri K., Morales M.L.V., Saad S.M.I., Adorno M.A.T., Sakamoto I.K., Rossi E.A. (2014). Prebiotic effect of fructooligosaccharide in the simulator of the human intestinal microbial ecosystem (SHIME (R) Model). J. Med. Food.

[140] Van Den Abbeele P., Venema K., van de Wiele T., Verstraete W., Possemiers S. (2013). Different human gut models reveal the distinct fermentation patterns of arabinoxylan versus inulin. J. Agric. Food Chem..

[141] Ouwehand A., Derrien M., de Vos W., Tiihonen K., Rautonen N. (2005). Prebiotics and other microbial substrates for gut functionality. Curr. Biol..

[142] Patterson J.A., Burkholder K.M. (2003). Application of prebiotics and probiotics in poultry production. Poult. Sci..

[143] Annison G., Illman R., Topping D. (2003). Acetylated, propionylated or butyrylated starches raise large bowel short-chain fatty acids preferentially when fed to rats. J. Nutr..

[144] Baurhoo B., Letellier A., Zhao X., Ruiz-Feria C.A. (2007). Cecal populations of Lactobacilli and Bifidobacteria and *Escherichia coli* after In Vivo *Escherichia coli* challenge in birds fed diets with purified lignin or mannanoligo-saccharides. Poult. Sci..

[145] Olveira G., González-Molero I. (2016). An update on probiotics, prebiotics and symbiotics in clinical nutrition. Endocrinol. Nutr..

[146] Sáez-Lara M.J., Robles-Sanchez C., Ruiz-Ojeda F.J., Plaza-Diaz J., Gil A. (2016). Effects of probiotics and synbiotics on obesity, insulin resistance syndrome, type 2 diabetes and non-alcoholic fatty liver disease: A review of human clinical trails. Int. J. Mol. Sci..

[147] Van Loo J., Clune Y., Bennett M., Collins J.K. (2005). The SYNCAN project: Goals, set-up, first results and settings of the human intervention study. Br. J. Nutr..

[148] Schiffrin E.J., Kumar V.B., Brown C., Hager C., Van’t Hof M.A., Morley J.E., Guigoz Y. (2007). Systemic inflammatory markers in older persons: The effect of oral nutritional supplementation with prebiotics. J. Nutr. Health Aging.

[149] Vulevic J., Drakoularakou A., Yaqoob P., Tzortzis G., Gibson G.R. (2008). Modulation of the fecal microflora profile and immune function by a novel transgalactooligosaccharide mixture (B-GOS) in healthy elderly volunteers. Am. J. Clin. Nutr..

[150] Schley P.D., Field C.J. (2002). The immune-enhancing effects of dietary fibres and prebiotics. Br. J. Nutr..

[151] Grajek W., Olejnik A., Sip A. (2005). Probiotics, prebiotics and antioxidants as functional foods. Acta Biochim. Pol..

[152] Gibson G.R., Wang X. (1994). Regulatory effects of bifidobacteria on the growth of other colonic bacteria. J. Appl. Microbiol..

[153] Bovee-Oudenhoven I.M.J., Termont D.S., Heidt P.J., van der Meer R. (1997). Increasing the intestinal resistance of rats to the invasive pathogen *Salmonella enteritidis*: Additive effects of dietary lactulose and calcium. Gut.

[154] De Preter V., Hamer H.M., Windey K., Verbeke K. (2011). The impact of pre- and/or probiotics on human colonic metabolism: Does it affect human health?. Mol. Nutr. Food Res..

[155] Demigné C., Jacobs H., Moundras C., Davicco M.J., Horcajada M.N., Bernalier A., Coxam V. (2008). Comparison of native or reformulated chicory fructans, or non-purified chicory, on rat cecal fermentation and mineral metabolism. Eur. J. Nutr..

[156] Mojka K. (2014). Probiotyki, prebiotyki i synbiotyki—Charakterystyka i funkcje. Probl. Hig. Epidemiol..

[157] Socha P., Stolarczyk M., Socha J. (2002). Wpływ probiotyków i prebiotyków na gospodarkę lipidową. Pediatr. Współcz. Gastroenterol. Hepatol. Żyw. Dziecka.

[158] Asahara T., Nomoto K., Shimizu K., Watanuki M., Tanaka R. (2001). Increased resistance of mice to *Salmonella enteritica* serovar Typhymurium infection by synbiotic administration of bifidobacteria and transgalactosylated-oligosaccharides. J. Appl. Microbiol..

[159] Buddington K.K., Danohoo J.B., Buddington R.K. (2002). Dietary oligofructose and inulin protect mice from enteric and systemic pathogens and tumour inducers. J. Nutr..

[160] Cummings J.H., Macfarlane G.T. (2002). Gastrointestinal effects of prebiotics. Br. J. Nutr..

[161] Scheppach W., Weiler F. (2004). The butyrate story: Old wine in new bottles? Butyrate appears to be essential for a wide range of intestinal mucosal health benefits; however, the mechanisms behind this remain to be determined. Curr. Opin. Clin. Nutr. Metab. Care.

[162] Kim Y.S., Tsa O.D., Morita A., Bella A. (1982). Effect of sodium butyrate and three human colorectal adenocarcinoma cell lines in culture. Falk Symp..

[163] Munjal U., Glei M., Pool-Zobel B.L., Scharlau D. (2009). Fermentation products of inulin-type fructans reduce proliferation and induce apoptosis in human colon tumour cells of different stages of carcinogenesis. Br. J. Nutr..

[164] Verghese M., Rao D.R., Chawan C.B., Williams L.L., Shackelford L. (2002). Dietary inulin suppresses azoxymethane-induced aberrant crypt foci and colon tumors at the promotion stage in young Fisher 344 rats. J. Nutr..

[165] Taper H.S., Roberfroid M.B. (2002). Inulin/Oligofructose and anticancer therapy. Br. J. Nutr..

[166] Parnell J.A., Reimer R.A. (2009). Weight loss during oligofructose supplementation is associated with decreased ghrelin and increased peptide YY in overweight and obese adults. Am. J. Clin. Nutr..

[167] Luo J., van Yperselle M., Rizkalla S.W., Rossi F., Bornet F.R., Slama G. (2000). Chronic consumption of short-chain fructooligosaccharides does not affect basal hepatic glucose production or insulin resistance in type 2 diabetics. J. Nutr..

[168] Lu Z.X., Walker K.Z., Muir J.G., O’Dea K. (2004). Arabinoxylan fibre improves metabolic control in people with type II diabetes. Eur. J. Clin. Nutr..

[169] Garcia A.L., Otto B., Reich S.C. (2007). Arabinoxylan consumption decreases postprandial serum glucose, serum insulin and plasma total ghrelin response in subjects with impaired glucose tolerance. Eur. J. Clin. Nutr..

[170] Daubioul C.A., Horsmans Y., Lambert P., Danse E., Delzenne N.M. (2005). Effects of oligofructose on glucose and lipid metabolism in patients with nonalcoholic steatohepatitis: Results of a pilot study. Eur. J. Clin. Nutr..

[171] Bruzzese E., Volpicelli M., Salvini F., Bisceglia M., Lionetti P., Cinquetti M., Iacono G., Guarino A. (2006). Early administration of GOS/FOS prevents intestinal and respiratory infections in infants. J. Pediatr. Gastroenterol. Nutr..

[172] Costalos C., Kapiki A., Apostolou M., Papathoma E. (2008). The effect of a prebiotic supplemented formula on growth and stool microbiology of term infants. Early Hum. Dev..

[173] Scholtens P.A., Alliet P., Raes M., Alles M.S., Kroes H., Boehm G., Knippels L.M., Knol J., Vandenplas Y. (2008). Fecal secretory immunoglobulin A is increased in healthy infants who receive a formula with short-chain galacto-oligosaccharides and long-chain fructo-oligosaccharides. J. Nutr..

[174] Welters C.F., Heineman E., Thunnissen F.B., van den Bogaard A.E., Soeters P.B., Baeten C.G. (2002). Effect of dietary inulin supplementation on inflammation of pouch mucosa in patients with an ileal pouch-anal anastomosis. Dis. Colon Rectum.

[175] Lindsay J.O., Whelan K., Stagg A.J., Gobin P., Al-Hassi H.O., Rayment N., Forbes A. (2006). Clinical, microbiological, and immunological effects of fructo-oligosaccharide in patients with Crohn’s disease. Gut.

[176] Moro G., Arslanoglu S., Stahl B., Jelinek J., Wahn U., Boehm G. (2006). A mixture of prebiotic oligosaccharides reduces the incidence of atopic dermatitis during the first six months of age. Arch. Dis. Child..

[177] Arslanoglu S., Moro G.E., Boehm G. (2007). Early supplementation of prebiotic oligosaccharides protects formula-fed infants against infections during the first 6 months of life. J. Nutr..

[178] Azcarate-Peril M.A., Ritter A.J., Savaiano D., Monteagudo-Mera A., Anderson C., Magness S.T., Klaenhammer T.R. (2017). Impact of short-chain galactooligosaccharides on the gut microbiome of lactose-intolerant individuals. Proc. Natl. Acad. Sci. USA.

[179] Gourbeyre P., Denery S., Bodinier M. (2011). Probiotics, prebiotics, and synbiotics: Impact on the gut immune system and allergic reactions. J. Leukoc. Biol..

[180] De Vrese M., Schrezenmeir J., Stahl U., Donalies U.E.B., Nevoigt E. (2008). Probiotics, prebiotics and synbiotics. Food Biotechnology, Advances in Biochemical Engineering/Biotechnology.

[181] Scavuzzi B.M., Henrique F.C., Miglioranza L.H.S., Simão A.N.C., Dichi I. (2014). Impact of prebiotics, probiotics and synbiotics on components of the metabolic syndrome. Ann. Nutr. Disord. Ther..

[182] Hamasalim H.J. (2016). Synbiotic as feed additives relating to animal health and performance. Adv. Microbiol..

[183] Sekhon B.S., Jairath S. (2010). Prebiotics, probiotics and synbiotics: An overview. J. Pharm. Educ. Res..

[184] Manigandan T., Mangaiyarkarasi S.P., Hemaltha R., Hemaltha V.T., Murali N.P. (2012). Probiotics, prebiotics and synbiotics—A review. Biomed. Pharmacol. J..

[185] Pandey K.R., Naik S.R., Babu V.V. (2015). Probiotics, prebiotics and synbiotics—A review. J. Food. Sci. Technol..

[186] Zhang M.M., Cheng J.Q., Lu Y.R., Yi Z.H., Yang P., Wu X.T. (2010). Use of pre-, pro-and synbiotics in patients with acute pancreatitis: A meta-analysis. World J. Gastroenterol..

[187] Wong V.W., Won G.L., Chim A.M., Chu W.C., Yeung D.K., Li K.C., Chan L. (2013). Treatment of nonalcoholic steatohepatitis with probiotics. A proof-of-concept study. Ann. Hepatol..

[188] Eslamparast T., Poustchi H., Zamani F., Sharafkhah M., Malekzadeh R., Hetmatdoost A. (2014). Synbiotic supplementation in nonalcoholic fatty liver disease: A randomized, double-blind, placebo-controlled pilot study. Am. J. Clin. Nutr..

[189] Pathmakanthan S., Walsh M., Bengmark S. (2002). Efficacy and Tolerability Treating Acute Distal Ulcerative Colitis with Synbiotic Enemas: A Pilot Trial (Abstract).

[190] Pérez-Conesa D., López G., Abellán P., Ros G. (2006). Bioavailability of calcium, magnesium and phosphorus in rats fed probiotic, prebiotic and synbiotic powder follow-up infant formulas and their effect on physiological and nutritional parameters. J. Sci. Food Agric..

[191] Danq D., Zhou W., Lun Z.J., Mu X., Wanq D.X., Wu H. (2013). Meta-analysis of probiotics and/or prebiotics for the prevention of eczema. J. Int. Med. Res..

[192] Rafter J., Bennett M., Caderni G., Clune I., Hughes R., Karlsson P.C., Klinder A., O’Riordan M., O’Sullivan G., Pool-Zobel B. (2007). Dietary synbiotics reduce cancer risk factors in polypectomized and colon cancer patients. Am. J. Clin. Nutr..

[193] Sanchez M., Darimont C., Drapeau V., Emady-Azar S., Lepage M., Rezzonico E., Ngom-Bru C., Berger B., Philippe L., Ammon-Zuffrey C. (2014). Effect of *Lactobacillus* rhamnosus CGMCC1.3724 supplementation on weight loss and maintenance in obese men and women. Br. J. Nutr..

[194] Safavi M., Farajian S., Kelishadi R., Mirlohi M., Hashemipour M. (2013). The effects of synbiotic supplementation on some cardio metabolic risk factors in overweight and obese children: A randomized triple-masked controlled trial. Int. J. Food Sci. Nutr..

[195] Ipar N., Aydogdu S.D., Yildirim G.K., Inal M., Gies I., Vandenplas Y., Dinleyici E.C. (2015). Effects of symbiotic on anthropometry, lipid profile and oxidative stress in obese children. Benef. Microbes.

[196] Eslamparast T., Zamani F., Hekmatdoost A., Sharafkhah M., Eghtesad S., Malekzadeh R., Poustchi H. (2014). Effects of synbiotic supplementation on insulin resistance in subjects with the metabolic syndrome: A randomised, double-blind, placebo-controlled pilot study. Br. J. Nutr..

[197] Asemi Z., Zohreh Z., Shakeri H., Sima-sadat Sabihi S.S., Esmaillzadeh A. (2013). Effect of multispecies probiotic supplements on metabolic profiles, hs-CRP, and oxidative stress in patients with type 2 diabetes. Ann. Nutr. Metab..

[198] Tajadadi-Ebrahimi M., Bahmani F., Shakeri H., Hadaegh H., Hijijafari M., Abedi F., Asemi Z. (2014). Effects of daily consumption of synbiotic bread on insulin metabolism and serum high-sensitivity C-reactive protein among diabetic patients: A double-blind, randomized, controlled clinical trial. Ann. Nutr. Metab..

[199] Shakeri H., Hadaegh H., Abedi F., Tajabadi-Ebrahimi M., Mazroii N., Ghandi Y., Asemi Z. (2014). Consumption of synbiotic bread decreases triacylglycerol and VLDL levels while increasing HDL levels in serum from patients with type-2 diabetes. Lipids.

[200] Moroti C., Souza Magri L.F., Rezende-Costa M., Cavallini D., Sivieri K. (2012). Effect of the consumption of a new symbiotic shake on glycemia and cholesterol levels in elderly people with type 2 diabetes mellitus. Lipids Health Dis..

[201] Šmid A., Strniša L., Bajc K., Vujić-Podlipec D., Bogovič Matijašić B., Rogelj I. (2016). Randomized clinical trial: The effect of fermented milk with the probiotic cultures *Lactobacillus acidophilus* La-5^®^ and *Bifidobacterium* BB-12^®^ and Beneo dietary fibres on health-related quality of life and the symptoms of irritable bowel syndrome in adults. J. Funct. Foods.

[202] Ustundag G.H., Altuntas H., Soysal Y.D., Kokturk F. (2017). The effects of synbiotic “*Bifidobacterium* lactis B94 plus Inulin” addition on standard triple therapy of Helicobacter pylori eradication in children. Can. J. Gastroenterol. Hepatol..

[203] Fujimori S., Gudis K., Mitsui K., Seo T., Yonezawa M., Tanaka S., Tatsuguchi A., Sakamoto C. (2009). A randomized controlled trial on the efficacy of synbiotic versus probiotic or prebiotic treatment to improve the quality of life in patients with ulcerative colitis. Nutrition.

[204] Van der Aa L.B., Heymans H.S.A., van Aalderen W.M.C., Sillevis Smitt J.H., Knol J., Ben Amor K., Goossens D.A., Sprikkelman A.B. (2010). Effect of a new synbiotic mixture on atopic dermatitis in infants: A randomized controlled trial. Clin. Exp. Allergy.

[205] Farid R., Ahanchian H., Jabbari F., Moghiman T. (2011). Effect of a new synbiotic mixture on atopic dermatitis in children: A randomized-controlled trial. Iran. J. Pediatr..

[206] Post J. (2013). Acceptability and Feasibility of Probiotic and Prebiotic Supplementation in Alleviating Symptoms of Lactose Maldigestion in Lactose Intolerant Subjects.

[207] Nowak A., Śliżewska K., Libudzisz Z. (2010). Probiotyki—Historia i mechanizmy działania. Żywność Nauka Technologia Jakość.

[208] Bomba A., Nemcova R., Mudronova D., Guba P. (2002). The possibilities of potentiating the efficacy of probiotics. Trends Food Sci. Technol..

